# Super-resolution microscopy with very large working distance by means of distributed aperture illumination

**DOI:** 10.1038/s41598-017-03743-4

**Published:** 2017-06-16

**Authors:** Udo Birk, Johann v. Hase, Christoph Cremer

**Affiliations:** 1 Superresolution Microscopy, Institute of Molecular Biology (IMB), D-55128 Mainz, Germany; 2 Physics Department University Mainz (JGU), D-55128 Mainz, Germany; 3 Kirchhoff Institute for Physics, University Heidelberg, D-69120 Heidelberg, Germany; 4 Institute of Pharmacy&Molecular Biotechnology (IPMB), University Heidelberg, D-69120 Heidelberg, Germany

## Abstract

The limits of conventional light microscopy (“Abbe-Limit“) depend critically on the numerical aperture (NA) of the objective lens. Imaging at large working distances or a large field-of-view typically requires low NA objectives, thereby reducing the optical resolution to the multi micrometer range. Based on numerical simulations of the intensity field distribution, we present an illumination concept for a super-resolution microscope which allows a three dimensional (3D) optical resolution around 150 nm for working distances up to the centimeter regime. In principle, the system allows great flexibility, because the illumination concept can be used to approximate the point-spread-function of conventional microscope optics, with the additional benefit of a customizable pupil function. Compared with the Abbe-limit using an objective lens with such a large working distance, a volume resolution enhancement potential in the order of 10^4^ is estimated.

## Introduction

Due to novel developments in optical technology and photophysics^1^ it has become possible to radically overcome the classical diffraction limit for high NA objective lenses (ca. 200 nm laterally, 600 nm along the optical axis; also called the Abbe-limit) of conventional far-field microscopy^2^. These discoveries which promise to revolutionize Biology and Medicine have been honored by the 2014 Nobel Prize in Chemistry to Eric Betzig and William Moerner, for developing single fluorophore detection as the basis for single molecule localization microscopy using photoactivated proteins; and to Stefan Hell for the development of Stimulated Emission Depletion (STED) Microscopy, a “focused nanoscopy” method^3^. Using these approaches, both optical resolution (smallest distance detectable between two adjacent point sources) and structural resolution (smallest structural detail determined based on the density of point sources resolved) has been enhanced very substantially. At the present state of the art, they allow a light-optical resolution of biostructures down to about 5 nm^4^, corresponding to 1/100th of the excitation wavelength λ_exc_.

However, due to the high NA objective lenses used in these studies, the thickness of an object which can be analyzed in 3D with such a high resolution in many approaches is presently restricted to a maximum of several tens of µm. This means that in most cases, only individual cells arranged in monolayers on glass substrates, or thin tissue sections can be studied at highest resolution.

For many biological and medical applications, this limitation of present super-resolution methods (SRM)^5, 6^ to a relatively small field of view, typically in the order of 100 µm diameter, and to thin objects poses a severe road block to developmental biology as well as to biomedical research: This limitation has hampered the full use of SRM methods to study e.g. the distribution of viruses, proteins or DNA/RNA sequences in three dimensional cellular arrangements, or to study microscopically disease correlated epigenetic changes on the single cell level in the organismic context. In many applications a field of view many times larger than 100 µm and a specimen thickness in the millimeter to centimeter range should be highly desirable^7^.

One solution for large field-of-view deep tissue imaging has been to design specialized objective lenses^8^ which implement a set of correction methods to compensate for aberrations. At a numerical aperture of NA = 0.47 and a working distance of 3 mm, it provides a field-of-view of ca. 6 mm across, thus allowing rapid data acquisition of large sample volumes. However, the lateral resolution is presently limited to ca. 1.3 λ (excitation wavelength in vacuum), and correspondingly the axial depth-of-focus is much larger than what can be obtained using high-NA objective lenses. In contrast to this existing system, illuminating the sample with light originating from an even larger solid angle (or a higher NA of the illumination scheme) would allow further reduction of the illumination spot. Another solution to study large fields of view of thin objects with high NA objective lenses has been to perform multiple acquisitions at different locations^9, 10^. For example, one might scan the object by multiple beams, e.g. 10,000 scanning beams, each scanning a field of view of 100 µm in diameter; in this case imaging could be parallelized, corresponding to a total field of view of 1 cm^2^. Such multiple beam scanning devices may be realized by using diffractive elements. In the case of Stimulated Emission Depletion microscopy (STED)^11^, steps in this direction have already been taken^10^, and one can obtain even super-resolution of extended thin objects in this way; also with diffraction limited beams like those used in confocal^12–14^ or 4Pi microscopy^15–17^ with laser excitation at constant intensity (continuous wave = cw), the combination with localization microscopy using photostable fluorophores has been experimentally realized^18–21^.

To obtain highly resolved images of thick fluorescence labelled objects like cellular aggregates, light sheet microscopy has been developed; with Bessel beam shaped light sheets^22, 23^, or in combination with axially structured illumination^24–28^; they already now provide a 3D resolution far beyond the conventional Abbe-limit. Such techniques are expected to be developed further and to be extended to still larger objects. In particular, light sheet microscopy of thick samples is designed to make use of a larger working distance thereby limiting the NA in detection for technical reasons^29^. Since the axial full-width-at-half-maximum (FWHM) is proportional to NA^−2^, the axial discrimination is severely impaired which is countered by the illumination with a thin light sheet aligned with the focal plane. Alternatively, enhanced optical resolution of thick 3D objects might be obtained by defined swelling of the biological structure^30^. This technique termed ‘expansion’ microscopy which acts on the sample side, rather than on the side of the instrumentation, may also be applicable to resolve subcellular or subnuclear biostructures. An additional way to achieve a substantial resolution enhancement would be the combination of structured illumination with optical projection tomography (OPT) approaches^31, 32^ or other forms of tomography^33–35^. In this way, it is anticipated to achieve single molecule resolution in an appropriately transparent, homogeneous object close to 1 mm in thickness.

In spite of these highly intriguing possibilities, a combination of “super-resolution” with still much larger working distances (e.g. 1 cm or more) appears to be extremely difficult to achieve with objective lens based microscopy devices. In this context, “super-resolution” would generally signify an optical resolution better than the Abbe/Rayleigh limit^36^ at the working distance given (e.g. 1 cm), using a single objective lens for imaging. For technical reasons, the NA of objective lenses with large working distance is limited: For high NA objective lenses, geometrical optics requires the radius of the optical lens (in practice the front lens) to be of the same order as the working distance^37^. Although solutions towards this end are being developed^8^, such large lenses are not only difficult (and expensive) to produce, but also difficult to house and to mount, and can not easily provide the same resolution obtained when using high-NA objective lenses. Secondly, application of immersion is not easily accomplished for large working distances as it would require the whole space between front lens and sample to be flooded with immersion, generally opposing the practical use of large working distances. Therefore, such large working distances are only possible with low or very low NA. For example, a working distance of 1 cm = 10 mm would require an objective lens with an NA of approximately 0.2 (assuming an air lens, n = 1). (Note that we use the distance between the illumination/detection optics and the sample as the definition of the working distance.) In theory, the sample could be brought in close proximity to the optics, and therefore imaging up to a depth corresponding to the working distance could be facilitated. In practice, aberrations due to the sample are likely to limit the imaging depth to a fraction of the working distance^38^. However, larger imaging depths have been shown to be feasible when using clearing protocols or highly transparent samples, potentially in combination with compensation for the introduced phase delay, see Supplementary Note 4). For such a low aperture (NA = 0.2) and assuming an imaging wavelength λ = 488 nm (vacuum wavelength; if not stated otherwise, λ = 488 nm will be used throughout the text), the Rayleigh formula would give an optical resolution of *d* = 0.61 λ/NA  =  1.5 µm laterally and about *d* = 2λ/(NA)² = 25 µm axially, resulting in an observation volume of the point-spread-function (PSF) of V_obs_ (NA = 0.2) = 4/3 π × 0.75 × 0.75 × 12.5 µm^3^ ≈ 30 µm^3^ assuming a Gaussian intensity distribution. (For simplicity of estimate, the FWHM of the PSF is assumed to be equal to *d*/2, i.e. V_obs_ = 4/3 π × FWHM_x_/2 × FWHM_y_/2 × FWHM_z_/2. For more correct estimates see Results). In the following, we shall use the term ‘observation volume’ for the smallest observable volume^39^; in the case of objective lens based imaging systems, this volume corresponds to the focal volume. (As the term ‘focal volume’ implicates the use of a focusing element, we refrain from its use in this report). For comparison, the observation volume now obtained in a commercial STED microscope in biological specimens for a high numerical aperture (NA = 1.4) is about V_obs_ (STED) = 4/3π × 0.03 × 0.03 × 0.3 µm^3^ = 0.001 µm^3^, or about 30,000 times smaller.

In principle, the typical resolution *d* in STED/RESOLFT microscopy is given by

1 $$d=\frac{{\lambda }_{exc}}{2n\,\sin (\alpha )\sqrt{1+{I}_{STED}/{I}_{sat}}}$$

where λ_exc_ is the fluorescence excitation wavelength, NA = *n* sin(*α*) is the numerical aperture (refractive index *n* and half-angle *α* of the light acceptance cone), *I*_STED_ is the intensity of the doughnut focused STED beam, and *I*_sat_ is the saturation intensity of the fluorophore used for STED imaging^40^. This formula predicts that it should be possible to achieve any STED resolution also at low NA (i.e. at large working distances) by an appropriate increase of the STED beam intensity; according to this relation, assuming the same wavelength and STED resolution, the required STED beam intensity scales inversely with NA^2^; this means that with an objective lens of numerical aperture NA = 0.2, an about 50 times higher STED beam intensity (1.4/0.2)^2^ would be required to achieve the same lateral resolution as with NA = 1.4; to what extent this will be practically possible and compatible with specimen conservation or live cell imaging is not known. Bleaching and phototoxicity already now appear to produce disadvantageous effects in many STED applications^41^; to overcome them at very large working distances probably would require the use of novel dyes with appropriately lowered saturation intensities *I*_sat_. Recent STED developments on making the depletion beam quasi-degenerate with the excitation beam in principle facilitate the operation at much lower disexcitation powers by using a depletion wavelength closer to the peak in the emission spectra^42^; nonetheless, for many dyes this advanced procedure is hampered by the increased cross-excitation due to the STED beam, resulting in a higher switching fatigue of the dye. Additionally, the localization precision in Single Molecule Localization Microscopy could be enhanced by using STED illumination^43^.

Alternatively, it remains highly desirable to consider the development of super-resolution techniques for very large working distances with substantially lower illumination intensities. Such techniques have been described for fluorescence microscopy approaches based on structured illumination with two excitation beams passing an objective lens^44, 45^; at a given numerical aperture, they provide an optical resolution enhanced by a factor two; in the example given above for NA = 0.2, this would result in a theoretical optical resolution of about 0.75 µm laterally and 12.5 µm axially; for NA = 0.1, the achievable lateral optical resolution would be d_SIM_ (NA = 0.1) = [0.61 λ/NA/2] = 1.5 µm. Proof-of-principle experiments^46^ using Retina cells with a structured illumination microscope featuring a working distance of about 4.5 cm indicated an optical resolution around 1.6 µm (as obtained from the spatial cut-off frequency), in accordance with the theoretical estimate.

The above mentioned theoretical and practical restrictions of optical resolution at large working distances are due to the low numerical aperture of the objective lenses used; however, these limits may be circumvented (i.e. the resolution can be enhanced many times more) by a scanning approach using a structured illumination concept with multiple beams focused constructively, thereby approximating the far-field of a spherical wave. The best approximation of the far field of a spherical wave is achieved in a “4π” geometry, which means that the light sources producing the individual beams are distributed over an area covering a full solid stereo angle of 4π as closely as possible. The basic idea to achieve SRM at large working distances by constructive focusing of multiple beams in such a 4π-geometry has been put forward already in the 1970s^13^; but so far numerical calculations of its feasibility have been lacking. In this report, we provide such numerical simulations; the results indicate that using an appropriate array of multiple collimated laser beams, an illumination focus with a Full-Width-at-half-Maximum (FWHM) around 140 nm in all directions can be produced (λ = 488 nm; n = 1.518) in a homogeneous, transparent medium. Since each of the coherent light beams is collimated, the distance of the sources is in principle arbitrary, i.e. this distance can be varied within large limits (e.g. up to several cm); this, however, is equivalent to the possibility to realize a joint ‘focal spot’ (similar to the illumination point-spread-function PSF_ill_ in conventional lens based illumination) for scanning based imaging. As discussed below, the joint ‘focal spot’ thus obtained can be made substantially smaller than possible with low NA objective lenses appropriate to realize the same large working distance; hence an enhanced resolution compared to the Rayleigh formula (using the same low NA) may be obtained.

Producing such a small focal diameter at a very large working distance is necessary in order to generate a strong signal response from within the object. But this is only the first requirement for enhanced resolution imaging: A second requirement is the detection of the generated signal, e.g. fluorescence or scattered light. Since at else equal conditions the fluorescence signal I_det_ detected is directly proportional to the area covered by the front lens of the detector system used, I_det_ scales inversely with the square of the working distance L. To give an example, if the photon flux entering the front lens of an NA = 1.4 objective at a working distance L_1.4_ = 0.2 mm is assumed to be I_flux1.4_, then for an equally sized front lens in the distance of L = 10 mm (assumed NA = 0.2) and the same refraction index, the photon flux I_flux0.2_ would scale inversely with L^2^ and hence be smaller by the factor [10/0.2]^2^ = 2500; and correspondingly we obtain for the localization accuracy σ_loc_ achievable in localization microscopy σ_loc_ ~ 1/N_det_^0.5^, where N_det_ = number of detected photons ~ I_flux_. As a consequence, the localization accuracy *σ*_*loc*_ would be 50 times worse, e.g. 1 µm nm instead of 20 nm, and the optical (two point) resolution would hence be around 2.35 µm (FWHM = 2.35*σ*_*loc*_) instead of around 50 nm. We shall discuss how to avoid such a deterioration of the fluorescence signal without having to sacrifice the advantages obtained by the small laser focus.

To produce a very small focal diameter for point-by-point-scanning of the object at a large working distance and to efficiently detect the generated signal (e.g. fluorescence or scattering) would already allow some highly interesting biophysical studies, e.g. to measure by Fluorescence Correlation Spectroscopy (FCS)^47^, the mobility and concentration of fluorophores in a very small cellular volume inside a large cellular aggregate, or a small model organism or entire organs (made suitably transparent). For example, using FCS at a large working distance with a numerical aperture of NA = 0.2 would monitor the fluorescence variation in an observation volume of V_obs_ = 30 µm^3^; in the 4π distributed aperture microscope (“4π-DAM”) described below, it should be possible to achieve at equivalent large working distances as for NA = 0.2 an estimated observation volume (for assumptions see above) around V_obs,4π_ = 4/3 × π × 0.07 × 0.07 × 0.07 µm^3^ ≈ 0.0014 µm^3^ i.e. many thousand times smaller. Another interesting application would be the possibility to introduce very small lesions inside a large cellular object, e.g. a chromatin damage inside a nucleus of a large cellular cluster; or to perform a corresponding optical stimulation e.g. of a neuron inside a thick sp﻿ecimen; or to facilitate the introduction of high resolution optical inspection into production lines.

To make possible imaging in the DAM, the object has to be scanned point-by-point with the ‘focal spot’ created. To realize this, either the beam has to be moved, or the specimen has to be moved. For simplicity, in this report we shall discuss only a stage scanning solution: Both the condition to move the stage and to optimize the fluorescence detection requires to use a beam array with some spacing between the beams; we shall present numerical calculations indicating that this requirement has only a slight effect on the achievable resolution.

As stated above, for the sake of simplicity of presentation, in the following conceptual study we typically assumed a vacuum excitation wavelength of λ = 488 nm and a refraction index n = 1.518. Similar to conventional imaging, the extent of the excitation spot within the precision of the calculations shown scales with λ/n; therefore other assumptions can easily be inferred from the example estimates shown.

## Results

**Figure 1:**
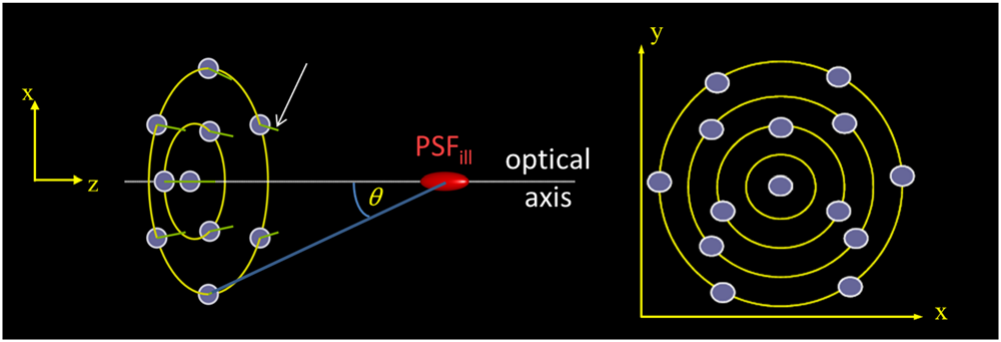
Schematic arrangement of light sources in the Distributed Aperture Microscope (DAM). Violet discs: Individual sources S_1_, S_2_, … S_K_, … S_N_ of coherent, collimated light at positions (x_K_, y_K_, z_K_) with defined phase and polarization relations emitting the light in defined directions (green); the red “spot” indicates the joint focal illumination distribution (i.e. the “focal volume” or the “observation volume of the illumination spot”) produced by the constructive interference of the collimated waves. Altogether, the sources span a solid angle ${\rm{\Omega }}=2\pi [1-\,\cos \,(\theta )]$, corresponding to the numerical aperture of a conventional objective lens. In such a configuration the Point-Spread-Function (PSF_ill_) of a conventional lens based illumination scheme may be approximated.

Figure 1 shows schematically the general arrangement to realize the multi beam illumination concept of DAM: A number (*N*) of point sources *S*_*i*_ (*i* = 1,2,3, … *N*) emitting collimated beams are arranged at defined positions ${{\boldsymbol{r}}}_{i}=({x}_{i},{y}_{i},{z}_{i})$ around the optical axis of the focusing DAM system. (Note that real illumination systems will often not provide truly collimated beams; instead Gaussian beam optics may be used to attribute for this deficiency; see Supplementary Notes 3 and 4.1). The following coordinate system is used: The geometrical center at which all sources are directed defines the origin O = (0,0,0) (red). The optical axis (*z*) is defined by the origin and the bary center of all sources. The spatial arrangement (i.e. the sites $({x}_{i},{y}_{i},{z}_{i})$ of the *N* light sources can be modified, covering a certain solid angle around the origin. The sources emit light in form of collimated beams (green, white arrow), directed towards the origin. The path length from all sources to the origin is equal (modulo λ, resulting in constructive interference such that the field in the origin is maximized. Thus the intensity distribution around the origin can be considered a ‘focal spot’. In this configuration, the plane perpendicular to the optical axis through the origin can be termed the focal plane.

### Calculation of known illumination distributions

**Figure 2:**
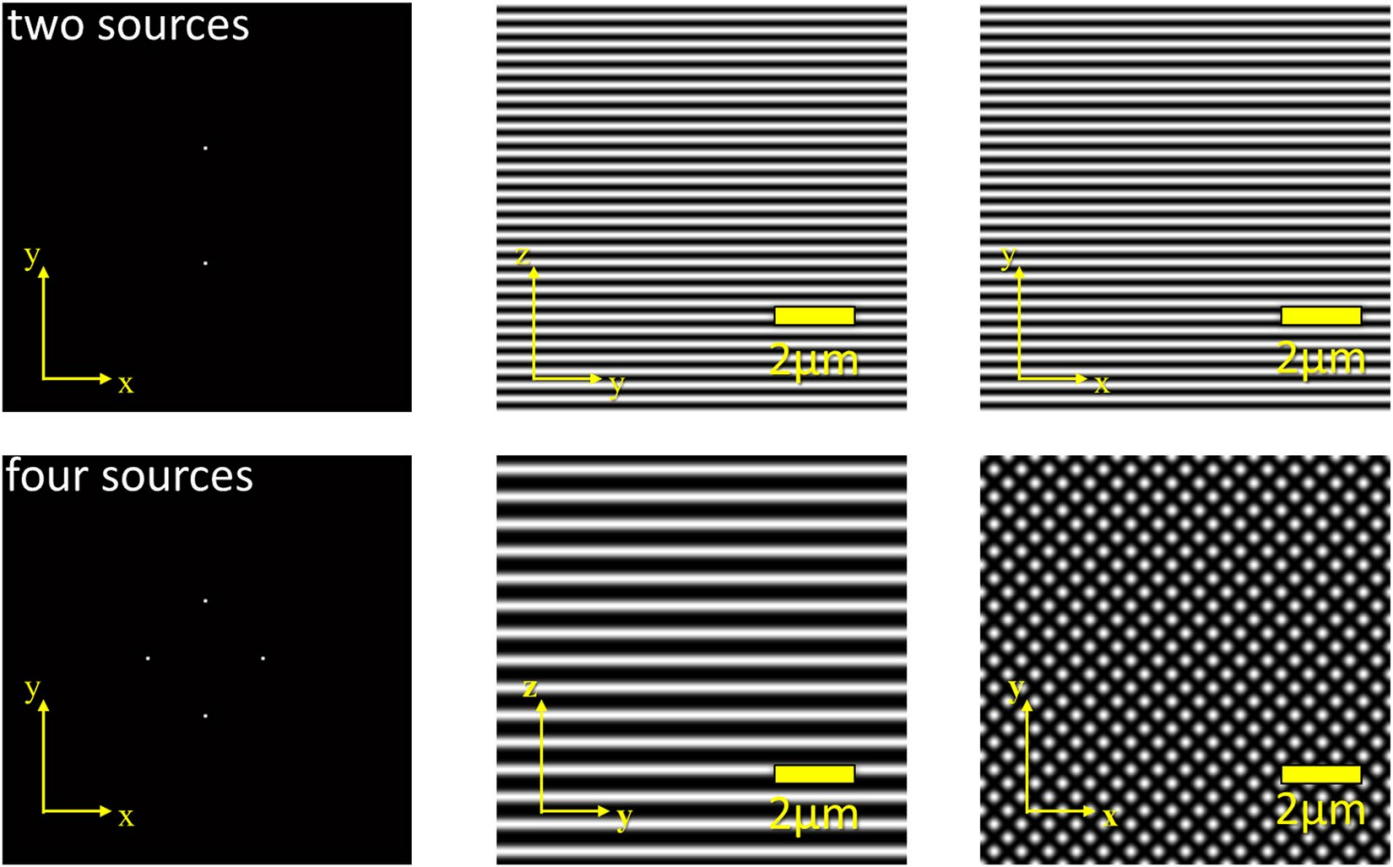
Arrangement of 2 and 4 coherent light sources and resulting focus intensity distribution. Left: z-projection of the arrangement of a few coherent light sources (e.g. glass fibers with low NA) directed towards the origin (refractive index n = 1). The optical axis is defined by the origin and the bary center of all sources. Sources are positioned at an elevation of 45° with respect to the optical axis. Center: y-z section across the intensity distribution around the origin. Right: x–y section across the intensity distribution around the origin.

To validate the correct implementation of the Feynman algorithm (see Materials and Methods and also Supplementary Information), we first calculated its predictions for a number of well-known optical conditions. For example, the case of two coherent light sources incident under an angle *θ* onto the focal plane corresponds to the concept employed in structured illumination microscopy, in which a single laser beam is split in two beams of equal intensity, and brought to interference in the object plane^48, 49^. This results in the formation of a standing wave along the object plane, with the direction of the modulation defined by the intersection with the plane through the optical axis and the two light sources (Fig. 2, top row). Interference of four beams arranged concentrically around the optical axis results in a 2D structured light field (Fig. 2, bottom row).

Intrinsically, the approach suggested by Feynman is of scalar nature, i.e. it neglects the polarization of the illumination sources. This leads to an error when calculating the interference pattern of light sources spanning a larger solid angle. Our computer simulations indicate that the results obtained when using the scalar approach are in good agreement with the results obtained using the full vectorial/electromagnetic description (see Supplementary Note 1).

### Generation of an illumination ‘focus’

**Figure 3:**
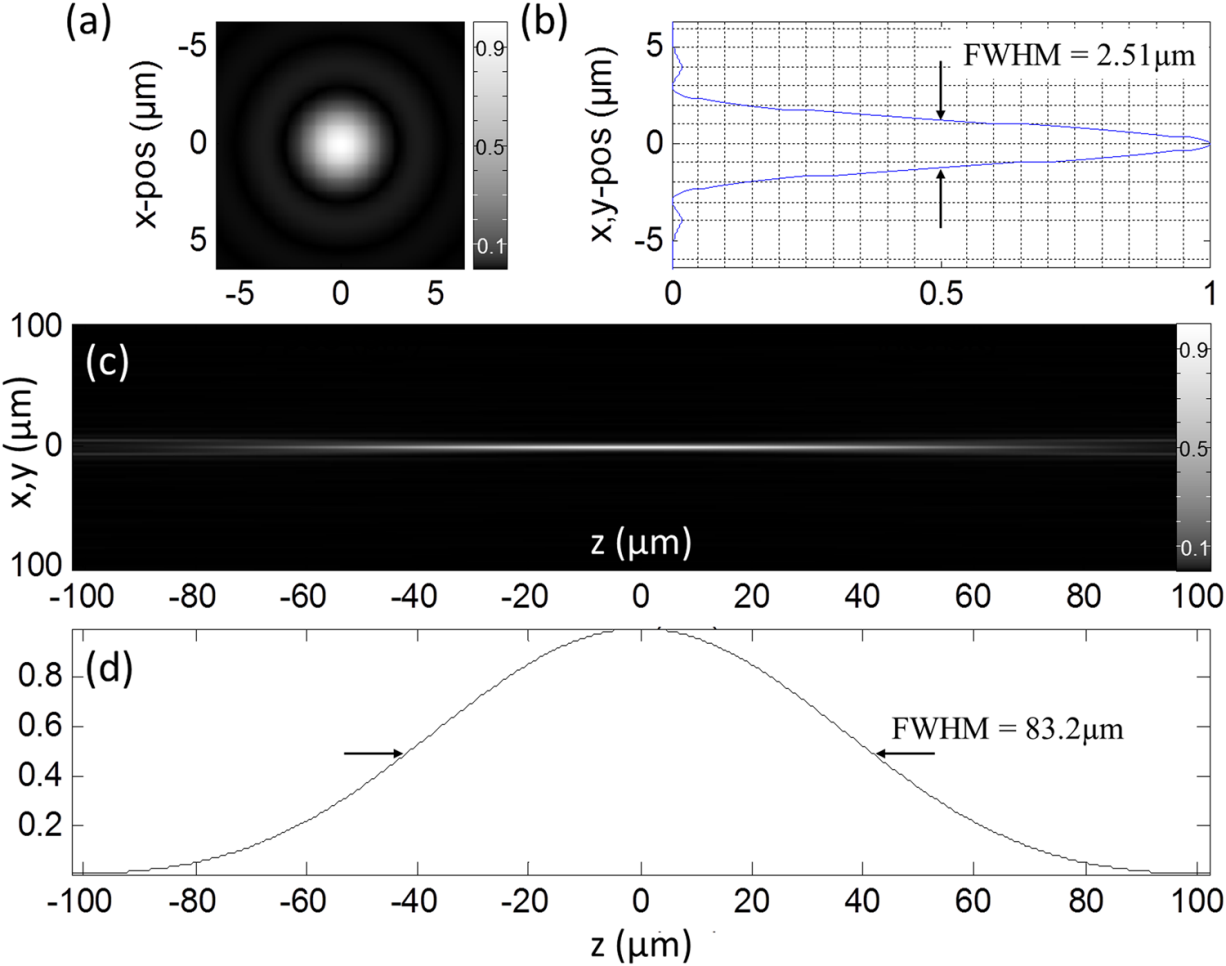
Focal spot for sources covering Ω = 0.01π. Intensity distribution when imaging with 6,499 sources of coherent photons distributed over a solid angle ${\rm{\Omega }}=2\pi (1-\,\cos \,\theta ) \sim 0.01\pi$ (this corresponds to an objective lens with NA = 0.1). Imaging media is air with refractive index n = 1.0. (**a**) Lateral distribution of the focal intensity F(x, y) (Gamma gray level scaling factor *γ* = 0.5). (**b**) Profile across the lateral focus intensity distribution along the y-axis. (**c**) x–z-section through the focus intensity distribution (*γ* = 0.5). (**d**) corresponding axial profile F_x=y=0_(z).

**Figure 4:**
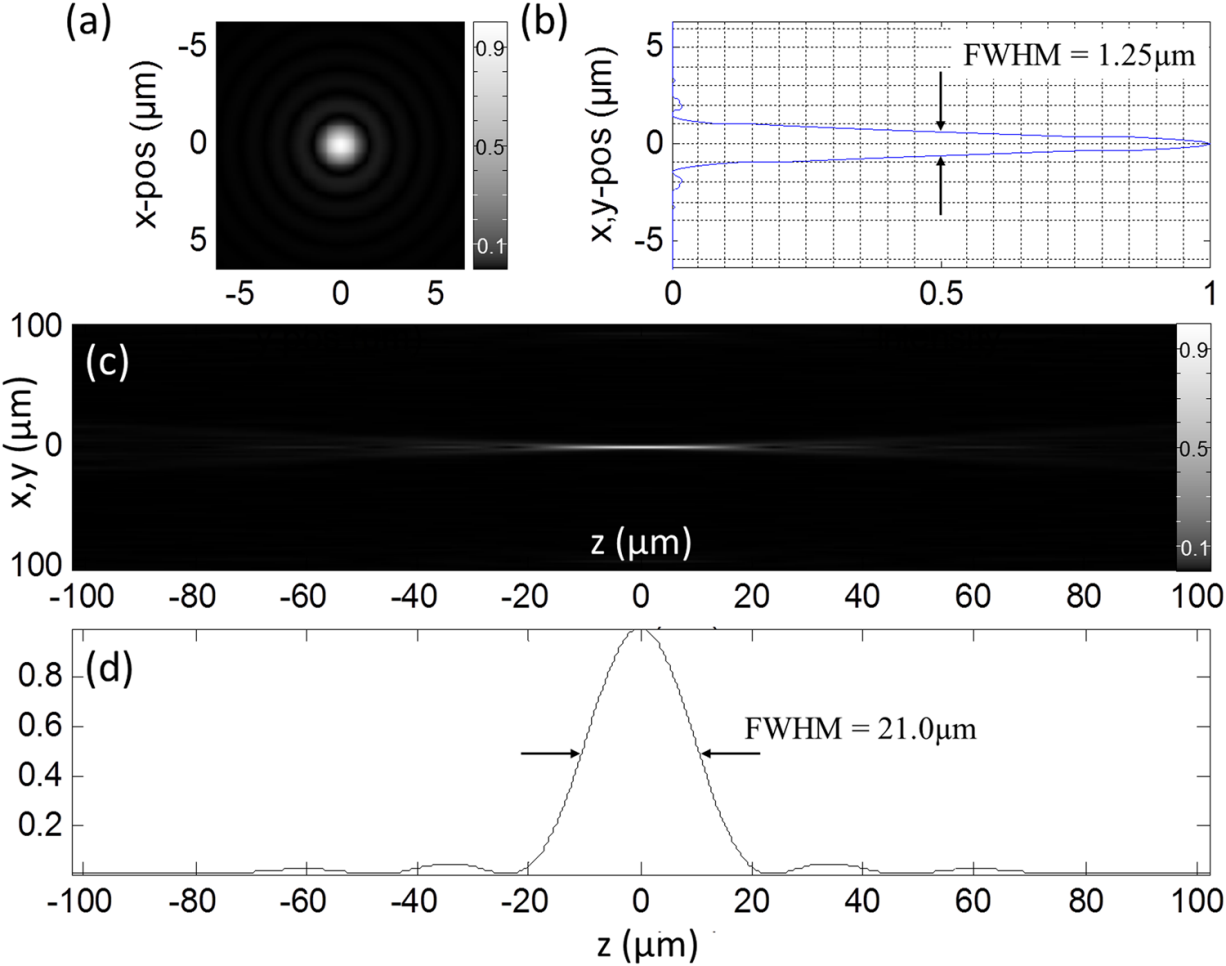
Focal spot for sources covering Ω = 0.04π. Intensity distribution when imaging with 6,482 sources distributed over ${\rm{\Omega }}=2\pi (1-\,\cos \,\theta ) \sim 0.04\pi$ (this corresponds to an objective lens with NA = 0.2). Imaging media is air with refractive index n = 1.0. (**a**) Lateral distribution of the focal intensity F(x,y) (*γ* = 0.5). (**b**) Profile across the lateral focus intensity distribution along the y-axis. (**c**) x-z-section through the focus intensity distribution (*γ* = 0.5). (**d**) Shows the corresponding axial profile F_x=y=0_(z).

**Figure 5:**
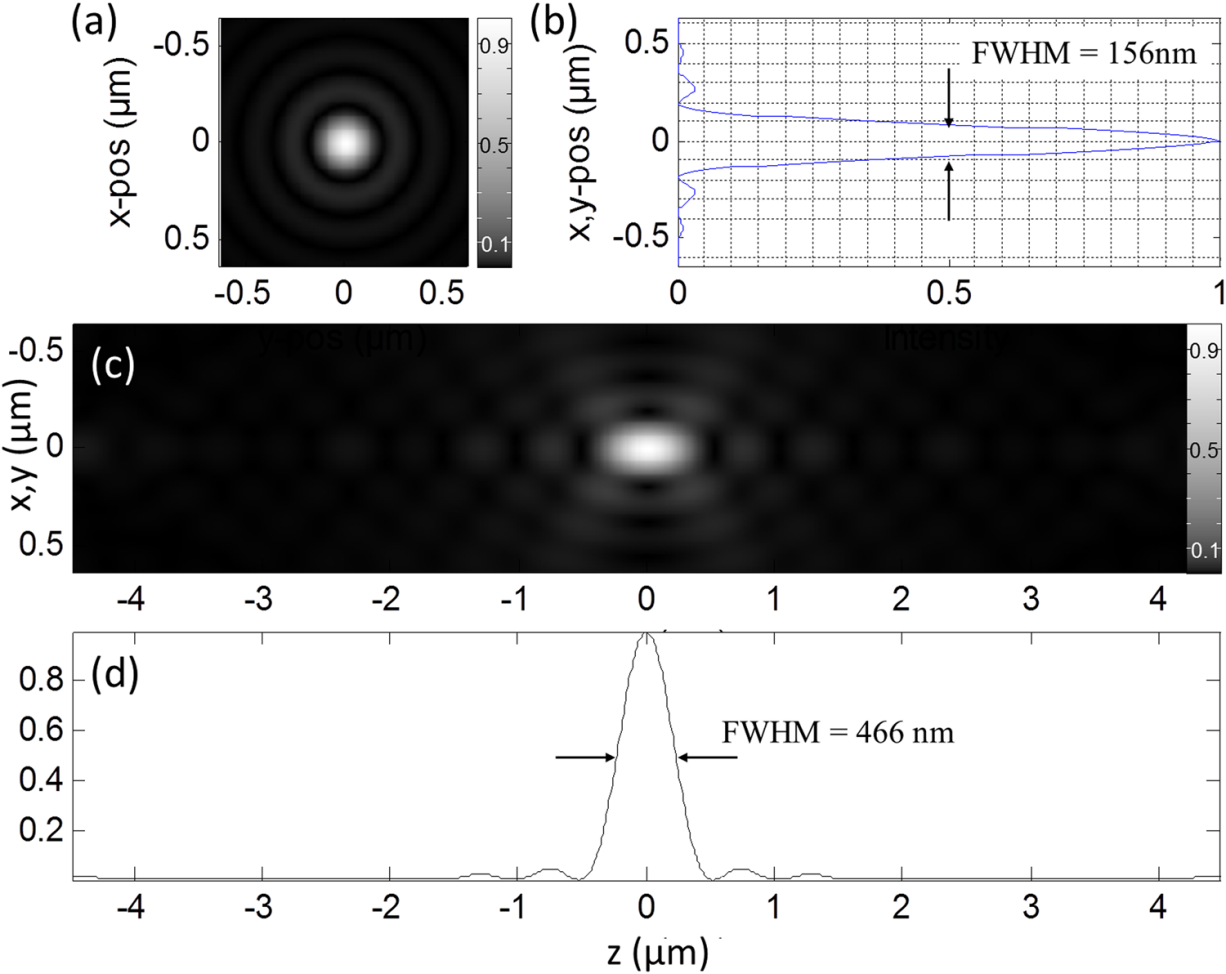
Focal spot for sources covering Ω = 1.25π. Intensity distribution when imaging with 6,580 sources distributed over ${\rm{\Omega }}=2\pi (1-\,\cos \,\theta ) \sim 1.25\pi$ (this corresponds to an objective lens with NA = 1.4). In this calculation, the refractive index at the position of the focus is given by n = 1.518. (**a**) Lateral distribution of the focal intensity F(x,y) (*γ* = 0.5). (**b**) Profile across the lateral focus intensity distribution along the y-axis. (**c**) x-z-section through the focus intensity distribution (*γ* = 0.5). (**d**) Shows the corresponding axial profile F_x=y=0_(z). In principle, the light source may be positioned at an arbitrary large distance from the focal region (also valid for Figs 3, 4 and 6).

To discriminate between lens based illumination and distributed aperture illumination, in the following *θ* is used to denote the maximum elevation of the sources (compare Fig. 1, while *α* is used for the half-angle of the acceptance cone when refering to conventional lens based illumination. Figures 3 and 4 show two examples of the application of Feynman’s algorithm to calculate the focal intensity distribution of approximately 6,500 sources distributed over a solid angle $\Omega =2\pi (1-\,\cos \,\theta ) \sim 0.01\pi$ (Fig. 3) and $\Omega  \sim 0.04\pi$ (Fig. 4). Similar focus intensity distributions may also be obtained by employing a long working distance objective lens with NA = 0.1 or NA = 0.2 respectively. In contrast to these results obtained for sources covering a relatively small solid angle, Fig. 5 shows the result of a number of beams approximating the focus of a high NA objective lens.

Considerations regarding the number of sources required to generate a suitable illumination spot are described in Supplementary Note 2. It was found that already with as little as *N* = 34 light sources distributed equally over the full solid angle of Ω = 4π, a minimum extent for the illumination spot of ca. 143 nm in all three spatial directions could be realized. The main formulae used to calculate the intensity distribution (using either scalar of vectorial/electromagnetic description) can be found in Supplementary Note 3.

In order to implement such an illumination scheme, physical light sources with defined phase, polarization, and direction have to be placed at defined positions around the conceptual center (of the illumination configuration), in which we aim to obtain maximum constructive interference i.e. a central illumination spot. Requirements towards the experimental realization of the illumination scheme in a real microscope setup are discussed in Supplementary Note 4. This includes a discussion of the physical representation of the light sources (Supplementary Note 4.1), the relation between the required working distance and the maximum number of sources (4.2), possibilities for sample mounting and detection of the fluorescence signal (4.3), the requirements towards the positioning of the light sources (4.4), as well as procedures for the alignment of the sources (4.5), adjustment of the phases (4.6), and operation of the instrument (4.7).

An inhomogeneous refractive index of the sample will result in a displacement of the beam axis at the target position (the origin); using a fiber based illumination approach, we expect that the beam waist has a diameter of approx. 0.5 mm (Supplementary Note 4.1B). As long as the displacement of the beam is less than approximately half this value, the criteria for constructive interference at this nominal target position can still be met by adjusting the phase of each individual light source. The net effect of the phase adjustment of the individual sources (Supplementary Notes 4.6 and 4.7) is the same as that introduced when using adaptive optics in conventional high-NA microscopy: The phase adjustment introduced to neighboring sources is an approximation to the phase-gradient in adaptive optics.

**Figure 6:**
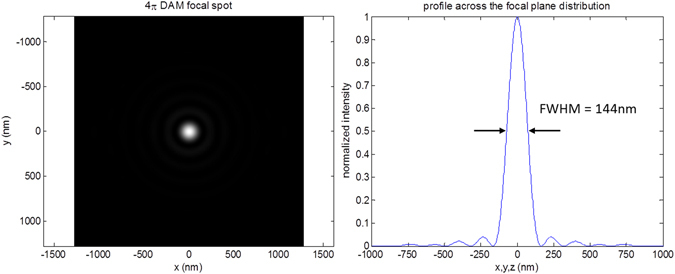
Focal spot for sources covering Ω = 4π (4π DAM). Intensity distribution when imaging with 9,016 sources distributed over ${\rm{\Omega }}=2\times 2\pi (1-\,\cos \,\theta ) \sim 4\pi$ (this cannot be realized with glass lenses, as it would theoretically correspond to two objective lenses each with NA = n sin(90°) = n, i.e. an acceptance cone with 180° opening angle). In this calculation, the refractive index at the position of the focus is given by n = 1.518. Left: Cross-section through the center of the light distribution in the focus. Right: Profile across the center of the light distribution in the focus. Note that for a sufficiently large number of equally distributed sources, the FWHM is equal in all three spatial directions (x, y, z).

Figure 6 presents the lateral and axial intensity plots through the focus calculated for a 4π microscopy arrangement realized by 9,016 sources equally distributed covering the full solid angle 4π. In practice, a central illumination spot of similar size as defined by the FWHMs in x, y, z can be realized with considerably less sources (Supplementary Note 2). In the experimental realization, it is unlikely that this resolution enhancement will be fully realised as any distortion of the beam due to the specimen will affect the achievable resolution. In conventional microscopy, such aberrations which are most noticeable at larger imaging depths^38^ are mitigated by the use of adaptive optics^50^. In this approach, the angular dependent phase delay introduced by the sample to the spherical wavefront cap is compensated by an active element such as a deformable mirror or a spatial light modulator. Similarly, the phase delay of the individual light sources introduced by the sample might be adjusted using a suitable calibration scheme (see Supplementary Notes 4.6 and 4.7). If successfully compensated, the theoretical values for the FWHM might be restored to a large degree in the experiment when imaging a real sample. However, even in this case we expect that best results will be obtained only after suitable clearing of the sample, i.e. after a sample preparation step with the aim to arrive at an approximately homogeneous refractive index.

**Figure 7:**
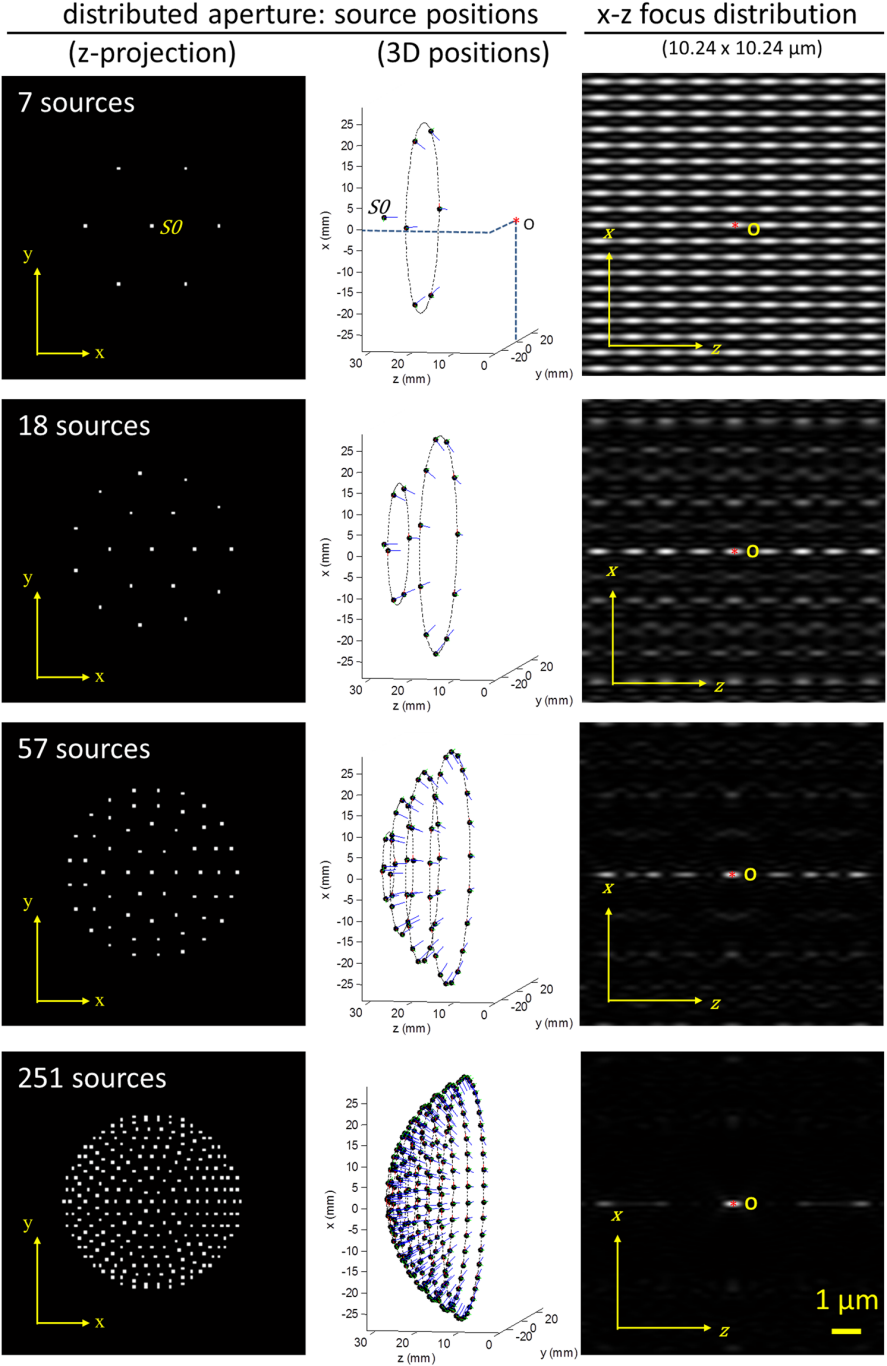
Arrangement of a number of coherent light sources and resulting focus intensity distribution. The light sources are arranged on a hemisphere such that the collimated light is directed towards the focal point at the origin O = (0, 0, 0). The sources have a distance d = 30mm from the origin. Note that the position coordinates can be multiplied by a constant scaling factor without changing the focusing properties as long as the beams remain collimated at the origin. Left column: z-projections of the source locations, with one central source S_0_ on the optical axis (z), and the other sources spread out to have approximately equal distances on the spherical surface. Center column: 3D plot of the source locations. The light sources are arranged on concentric rings (dashed lines) around the origin, with an increasing number of sources (n = 1, 6, 12, 24, 29, 34, 39, 42, 46) with increasing ring diameter (in this example up to 28 mm). The location of the light sources is indicated by black dots, the direction of the collimated beam is indicated by a blue line. Each light source is directed towards the origin. Right column: Resulting intensity distribution in the focal region as calculated using scalar theory (see Supplementary Note 1 for a quantitative assessment of the errors resulting from the use of scalar rather than vector theory).

Figure 7 shows the configuration employed to generate a focus intensity distribution with an increasing number of coherent light sources. The right column depicts the intensity distribution in the vicinity of the focus obtained for a distribution of sources spanning a solid angle corresponding to that of an objective lens with a numerical aperture NA = 1.4. For calculation, the spatial distribution of the light sources S_K_ was arranged in such a way that the solid angle covered by the sources covers the acceptance cone of an objective lens of NA = 0.2. The FWHMs of the ‘focal spot’ obtained with the Feynman algorithm fit well to the general expectation expressed by the Abbe-formula d_lateral_ = 0.5 λ/NA for the lateral focal diameter. Since in principle the light sources may be placed at any arbitrary large distance, this means that ideally also the working distance for the illumination may become arbitrarily large. In practice however, the distance of the light sources from the target position (origin) will typically not exceed a few centimeter.

**Figure 8:**
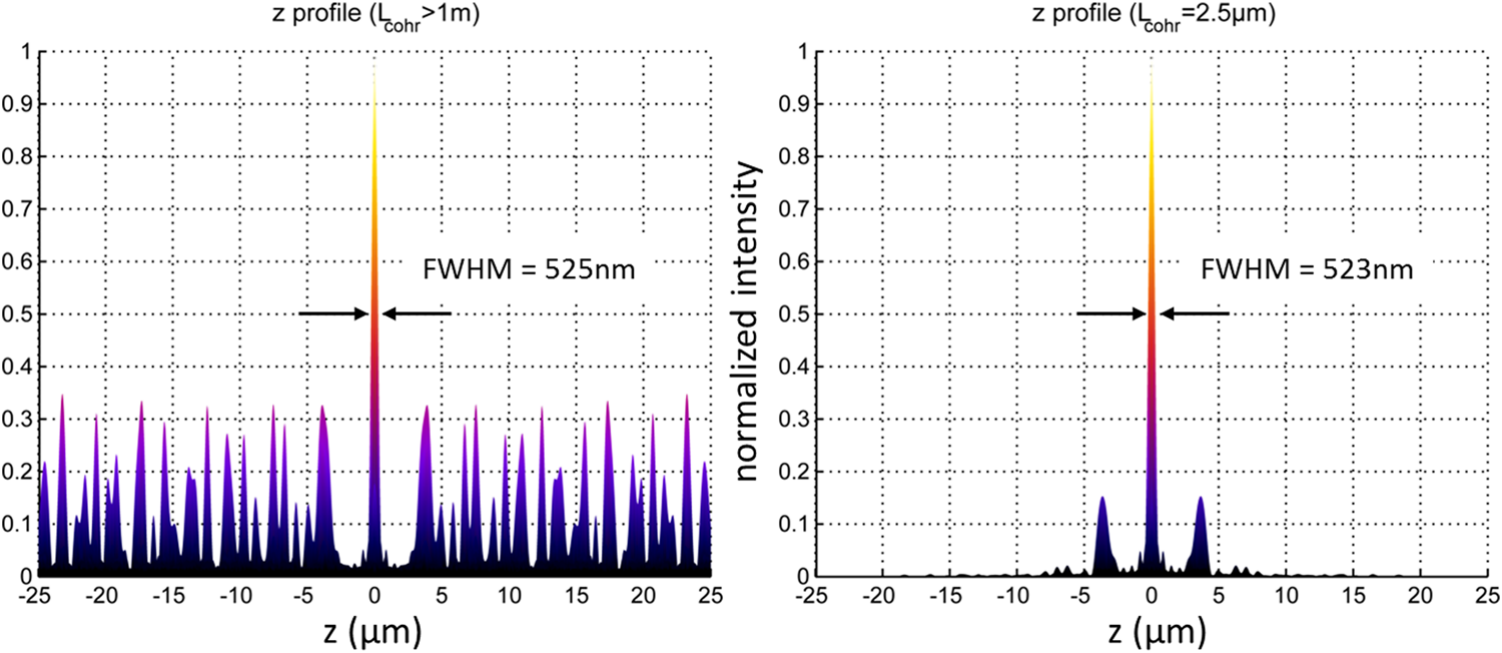
Suppression of side lobes. Side lobes in the vicinity of the focal point can be suppressed by means of low coherence length (L_cohr_) light sources. Calculation parameters: N = 251 sources, distributed within a solid angle of ${\rm{\Omega }}=2\pi (1-\,\cos \,\theta ) \sim 1.25\pi$ (this corresponds to an objective lens with NA = 1.4); λ = 550 nm; refractive index n = 1.518. Left: a light source with a coherence length larger than 1 m is used. Right: a light source with a coherence length of 2.5 µm is used. The relative height of the secondary maxima at ± 3.7 µm is reduced from 32.7% to 15.3%, allowing an appropriate resolution enhancement by deconvolution^51^ even at linear excitation.

Figure 7 clearly depicts one of the apparent disadvantages when approximating the spherical wavefront of a conventional objective lens with a relatively low number of collimated waves (e.g. 18 sources): The focus intensity distribution exhibits a repetitive pattern with secondary maxima placed at well-defined distances from the main focus. However, it is possible to make use of these additional foci: If the distance of the foci is larger than the resolution of the light detection system, the DAM may be equipped with a 2D detector array in addition to the beam scanning device. In such a configuration, the data acquisition speed is largely improved by simultaneously acquiring the fluorescence signal generated in several foci. In case that such a separation of neighboring foci cannot be achieved, or the generation of fluorescence signal from secondary maxima is otherwise undesirable, another solution exists. The DAM can be equipped with a light source of low coherence length. In such a case, the height of the secondary maxima can be dramatically reduced as indicated in Fig. 8. In this case, precise control of the absolute phases of the individual beams at the focus is essential. For light sources with a frequency band (indicative of a lower coherence length), controlling the phase relation over the whole frequency range might not be possible in a continuous wave illumination scheme. In such a case, the use of pulsed light source (with the potential for adding a pre-chirp^52^) could be a solution, but will be difficult to implement for a large number of point sources. The use of partially coherent illumination under a number of angles has previously been shown to allow holographic depth resolved imaging on a chip^53^.

### Concept for fluorescence depletion

**Figure 9:**
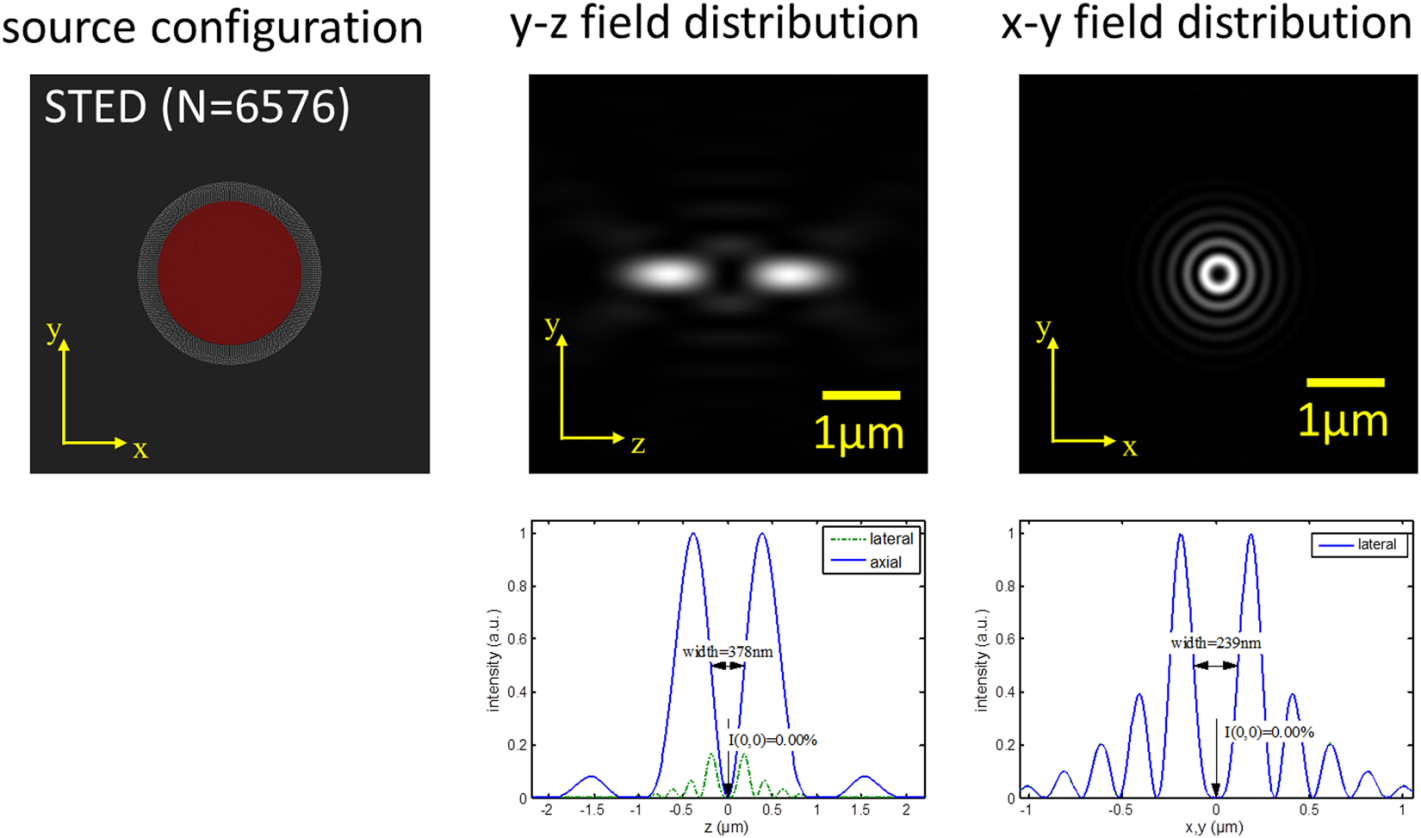
Concept for stimulated fluorescence emission depletion. Left: z-projection of the arrangement of N = 6,576 coherent light sources (e.g. glass fibers with low NA) directed towards the origin. Sources are distributed within a solid angle of ${\rm{\Omega }}=2\pi (1-\,\cos \,\theta ) \sim 1.25\pi$ (This corresponds to an objective lens with NA = 1.4). Center: y-z section across the intensity distribution around the origin. The width given (left: 378 nm, right: 293 nm) refers to the FWHM of the respective minimum (along the z- or x,y-axis). Right: x-y section across the intensity distribution around the origin. For STED-type illumination, half of the sources in the center were phase-delayed by $\pi$ (highlighted in red).

The concept of the distributed aperture illumination can readily be applied for generation of a depletion focus similar to that implemented in a STED microscope. The results of such calculations are depicted in Fig. 9. If such an intensity distribution be applied to a previously excited ensemble of fluorophores, the concept of fluorescence depletion in the vicinity of the origin can be realized at arbitrarily large working distances, without requiring the STED illumination intensities to be significantly higher than usually applied e.g. in commercial STED systems. It has not escaped to the notice of the authors, that in this way, a STED resolution similar to Equation (1) may be realized at working distances in the mm to cm range or even beyond using similar STED intensities as in high NA systems.

In a real experimental setup, alignment of several thousand individual illumination beams (phase, direction, intensity, collimation, polarization) is not practical. As discussed above, a well-defined illumination spot with narrow FWHM can be obtained using a much lower number of sources (see Supplementary Note 2). Likewise, a STED depletion pattern can be realized by illuminating the sample using three standing wave patterns (i.e. six coherent sources) with perpendicular polarization, effectively generating a 3D donut mode in the 4Pi-DAM system (see Supplementary Note 5).

## Discussion

### Illumination

The lateral and axial intensity plots through the focus were calculated for an arrangement of the light sources S_K_ covering the same solid angle as that of an objective lens with known numerical aperture (NA = 0.1, 0.2, 1.4). The FWHMs obtained with the Feynman algorithm (see Materials and Methods) fit well to the electromagnetic calculations by Hell and Stelzer^16^. The differences in the results for the two approaches are discussed in Supplementary Note 1. The remaining differences for a large number of sources may be attributed to the different assumptions made (e.g. finite number of sources emitting collimated beams instead of a virtually infinite number of spherical waves according to the Huygens principle).

The decisive difference of a microscope using a finite number of coherent sources for illumination of the sample to the application of conventional objective-lens-based microscopy, is the possibility to realize a substantially increased working distance. Two major benefits can potentially result from a larger working distance: First, there is much less chance of contamination of the sample in case where such a requirement is present, e.g. in clean rooms, in the hospital, in the food industry, material sciences, etc. Second, in case the refractive index of the sample can be made sufficiently homogeneous, e.g. by clearing the samples, there is the potential benefit of imaging deeper into thick objects without having to sacrifice resolution: While in conventional 4Pi microscopy and present commercial high NA STED microscopy the working distance has been limited to the 170 μm range and the maximum object thickness to values far below 100 μm at least for the 4Pi microscope, in principle in the DAM case any working distance can be chosen arbitrarily (e.g. 1 cm or even more), as long as the scattering within the sample is negligible and compensation for phase delays^32^ can be introduced (see Supplementary Notes 4.6 and 4.7). Due to the fact that the resolution value (FWHM) of the detection light path alone will be much larger, in such scanning based microscopy approaches, the resolution is primarily determined by the extent of the distribution of the illumination intensity in the focal plane, i.e. in the plane defined by the detection optics. The reason for this is that the detection probability can be considered to be more or less constant over the extent of the illumination spot. Therefore in principle, an increased high working distance does not result in a decreased resolution.

For example, if one compares the focal diameter calculated for the 4π-DAM arrangement with the focal diameter obtained by a NA = 0.2 lens, the lateral 4π-DAM diameter (FWHM_xy_ of the PSF_ill_) is an order of magnitude smaller (1250 nm/140 nm = 9, compare Figs 4b and 6); for the axial diameter (FWHM_z_) the effect is even larger as they differ by a factor 150 (0.14 μm for FWHM_z,4π-DAM_ vs. 21 μm for FWHM_z,NA0.2_, compare Fig. 4d). In terms of the 3D observation volume, the difference (using the numerical values of Figs 3 and 6) would be around V_obs,NA0.2_/V_obs,4π-DAM_ = (0.625 × 0.625 × 10.5 μm^3^)/(0.07 × 0.07 × 0.07μm^3^) = 1 × 10^4^_._ In a practical application, the detection efficiency of the distributed aperture configuration at large working distance will be limited, therefore reducing the signal-to-noise ratio of the detected fluorescence signal. This in turn will also affect the achievable resolution.

A major advantage of the DAM concept is its flexibility. The use of individual sources in principle allows one to adjust the amplitude, phase, direction, and polarization for each light source individually. As a consequence, the focus intensity distribution may be altered according to the specific needs. Possibly, also the collimation can be adjusted individually, e.g. via fast scanning liquid lenses^54^. In principle, adjustment of these source parameters may be used to steer the position of the focal spot, thereby implementing beam scanning. Furthermore, compensation of the aberrations in high-NA imaging systems^55^ becomes possible, as in principle any apodization function can be synthesized.

In the 4π-DAM concept presented, a solid angle of up to 4π has been assumed for the illumination scheme. By this, a small ‘focal spot’ diameter (illumination PSF) can be achieved in all spatial directions. In many cases, it may be advantageous to perform line-scanning or plane scanning instead of point-scanning. This should be possible by arranging the collimated beams in a plane. In this case, similar to structured illumination microscopy, the enhanced resolution image would be obtained by the combination of e.g. line scanning images from different directions (e.g. by rotating the object by an angle φ). Using appropriately collimated illumination sources, the generation of Bessel beams such as those used in Bessel beam light sheet microscopy with the additional possibility of introducing illumination lattices should also be possible.

### Detection

To register the fluorescence generated by focusing the laser beams in the DAM, an appropriate detection system is required. In the following discussion, a working distance in the 1 cm range will be assumed, as this appears to be adequate for many practical applications (see above). In principle, a lens of numerical aperture 0.1 or 0.2 would be sufficient for detection of the fluorescence signal. Such a lens can be placed in the working distance required (see Supplementary Note 4.3). However, the signal-to-noise ratio would be drastically reduced as compared to detection with a high-NA objective lens, possibly requiring the use of additional background signal suppressing hardware (Supplementary Note 4.3). For registration of scattered light (rather than fluorescence), a reduced detection efficiency is less of an issue. However, the amplitude of the scattered light depends strongly on the direction between illumination and detection. In this case, it might be preferable to use subsets of point sources for illumination covering a small solid angle, and to compose the final reconstructed image from the light scattered under various angles^56^.

Instead of using a low numerical aperture objective lens for fluorescence detection of the ‘focal spot’ produced by the 4π-DAM, one might also think of an arrangement of glass fibers connected with a point detector. Such an arrangement has already been proposed in the original 4π concept^13^. However, we assume that the collection efficiency using a fiber based detection system will be limited by the losses due to the fiber coupling and the limited coverage of the full solid angle over which the fibers are positioned, even if equipped with additional collection optics. Possibilities for a lens based detection scheme are discussed in Supplementary Note 4.3.

In the “proof-of-principle” calculations presented, stage scanning was assumed for point-by-point scanning and generation of 3D images. However, it is anticipated that by appropriate adjustment of the illuminating light sources, it should be possible to perform the scanning process also by beam scanning and thus highly accelerate the imaging process.

Finally, one has to consider that in a large object with widely distributed fluorescence emitters (e.g. a cellular spheroid with GFP labelled histones, or immunolabelled receptors), the multiple beams will excite also fluorophores outside the focal area; this emission may become so strong that the fluorescence produced at the focal site may not be clearly distinguished from the out-of-focus fluorescence (“background”). A well-known way to overcome this problem in conventional, lens-based microscopy is the use of two-photon excitation. Before such a solution could be realized in the 4π-DAM-system, additional technical problems related to the beam adjustment would need to be solved, which are mainly due to the restricted coherence lengths of short laser pulses. Due to the larger wavelength of two-photon excitation, it would additionally reduce the achievable optical resolution: for example, assuming two-photon excitation by 700 nm femtosecond pulses instead of using 488 nm, the optical resolution will be reduced by the factor 700/488 ≈ 1.4. But since the same requirement would also hold for the fluorescence imaging using a low aperture objective lens, it would not change the large relative resolution enhancement possible in the 4π-DAM at very large working distances.

The use of coherent light sources in principle permits the implementation of holographic detection. In particular, one interesting application for this interferometric imaging is microscopy, in which synthetic-aperture generation permits the extraction of medium- and high-resolution images using low-NA microscope lenses^57^. In contrast to the proposed illumination scheme using coherent light sources, incoherent illumination of semi-transparent samples has been shown to allow quantitative phase retrieval using quadriwave lateral shearing interferometry^58^. In combination with a number of polarization sensitive detection, the DAM could possibly be extended for multiplexing ellipsometric measurements.

## Conclusion

The numerical calculations presented provide further evidence that the old idea to realize a high-resolution laser scanning microscope with a very large working distance by using the constructive interference of multiple laser beams in a full “4π solid angle“ arrangement^13^ is indeed feasible from the point of Physics. This means that in this way super-resolution imaging can be achieved in the general sense, i.e. that at a given NA (for each single illumination or detection light path) the resolution can be substantially enhanced compared with the limit postulated by the Abbe/Rayleigh formula at the same NA. In combination with a suitable non-linear response excitation scheme, such as those implemented in e.g. STED microscopy, even super-resolution imaging in its strict sense becomes possible (i.e. resolution beyond the conventional resolution when using **high-NA** objective lenses). It is obvious that the realization of such a concept will require major efforts from the side of optical technology. It is anticipated that the possibility of the DAM to adjust the intensity, phase, polarization and direction of each beam individually will allow the implementation of an adaptive optics feedback system in order to diminish such problems. If successful, such approaches making use of the full solid angle will allow us to obtain spatial information from selected sites in transparent thick objects of homogeneous refractive index, or in thin objects at large working distance, at an unprecedented level of optical resolution.

## Material and Methods

### Concept for a multi beam illumination setup

In the original concept of a confocal laser scanning 4π fluorescence microscope enabling “super-resolution” with a large working distance^13^, point-by-point excitation of the object in 3D was assumed to be possible by the use of “4π holograms” to focus the incident coherent light beams to a focal “spot” with a diameter smaller than possible by focusing through a single lens. In the distributed aperture microscopy (DAM) concept presented here, a number *N* (from a few to several thousand) of coherent, continuously emitting point sources *S*_1_, *S*_2_, …, *S*_*N*_ are positioned at coordinates ***r***_1, … *N*,_ distributed around the object to be investigated. The point sources *S*_1, … *N*_ have fixed phase and polarization relations. For an arbitrary but fixed configuration (positions, phases, polarizations) of light sources, the origin of the coordinate system is placed at the theoretical absolute maximum of the illumination intensity distribution (i.e. the theoretical ‘focus’). The point of origin together with the bary center of the positions of the light sources defines a set of two positions in space; the line through both points constitutes the optical axis (*z*).

Generation of multiple coherent, collimated beams with fixed phase and polarization relation is possible in a number of ways by dividing light from a single laser light source e.g. by means of a microlens array, and guiding the individual beams to the desired positions of the point sources: a) using free space optics or b) using a glass fiber based approach (see Supplementary Notes 4.1–4.3). Such a microlens configuration should be used conjunctly with a liquid crystal array in transmission mode in order to provide control of the phase relation at the location of the point sources.

Implementation of a small number of sources has previously been investigated as a concept to enhance resolution^24^ (see also Supplementary Note 2). Such methods were termed excitation field synthesis. By combining images taken with a number of fine interference patterns superimposed on the object, so-called synthetic aperture microscopy could be realized^59^. Concepts which are similar in nature but which operate on the detection side have been applied for many years in astronomy. Such methods usually require the simultaneous use of two (or more) detection optics elements (i.e. detection lenses) in order to arrive at coherent constructive interference, as otherwise the signals captured in these multiple low NA images will not add up to a high NA image. For two high-NA objective lenses this has been realized in some of the 4Pi confocal laser scanning microscope systems (4Pi Type B with coherent detection).

The utilization of multiple collimated beams (Supplementary Note 4) has several advantages: (a) the use of collimated beams can be realized in principle by any distance of the light sources S_K_ emitting the collimated beams; (b) with increasing distance from the origin, the number of collimated beams can be made very large; (c) the number, intensity and spatial distribution of the light sources S_K_ (using e.g. microlenses) can be individually adjusted. For example, by adding a possibility to control the intensity I_K_ of the collimated beam emitted by a light source S_K_ (e.g. in a similar way as in a projection beamer), the light distribution can be modified individually in a fast and efficient way. When compared to conventional lens-based microscopy, this allows – in principle – direct access to and modification of the pupil function of the illumination lens.

### Calculation of the illumination intensity distribution

A comprehensive”classical method” to calculate the focusing of light in the context of the electromagnetic theory of waves has been described by^60^. Essentially, the solution is given by a number of Integrals I_0_ containing products of trigonometric functions, a Bessel function with a product of two trigonometric functions in the argument, and a complex exponential function with a product of two trigonometric functions as argument. The Bessel functions themselves are not elementary functions, i.e. they have to be given as numerical approximations. The same approach has been successfully applied to calculate very effectively the constructive focusing of coherent light in confocal laser scanning fluorescence microscopy as well as in super-resolving confocal laser scanning 4Pi microscopy using two high NA objective lenses^15, 16^, and experimentally measured also for other configurations of polarizations and apertures e.g. by Dorn *et al*.^61^.

While these integral solutions are highly elegant and have been shown to satisfactorily describe focusing by various arrays of glass lenses, it appears to be difficult to use them to calculate the focal intensity produced by a finite number of single collimated beams emitted by light sources placed at specific positions, with individually specified intensities and propagation directions. Therefore we used a more elementary and more flexible way of calculations, based on an idea by Richard Feynman^62^. In this approach, instead of waves, light propagation is considered as a flow of photon ‘particles’ (see also Supplementary Note 3). The electromagnetic wave at any given site (x, y, z) is the vector-sum of a suitably defined photon flux, and the intensity is correspondingly given by the sum-squared over the orientation of polarizations. The squareroot of the probability to detect these photons at a given site (x, y, z) is described by the sum of probability vectors A, where the direction of A denotes the phase difference compared to a probability vector along the perpendicular abscissa of the plane assumed to have zero phase, and the length of A denotes its probability amplitude; i.e. the square A^2^ gives the probability P to detect photons at the site indicated by the vector A. Apart from a normalization, A^2^ is the averaged number of photons detected per units of time and area perpendicular to the k vector (k = 2π/λ), i.e. the light intensity observed at a given site. If different A vectors are summed up (corresponding to the superposition of different waves with different phases), the squared length A_res_^2^ gives the intensity at the site considered.

To sum up waves, it is assumed that the polarization of light has a given direction for each beam; the direction of the polarization, however, may be changed according to the specific light beam considered; the wave amplitude or resultant polarization at a given site is obtained from appropriate summing up the probability vectors having different polarization. This can be achieved by appropriate vector addition rules.

**Figure 10:**
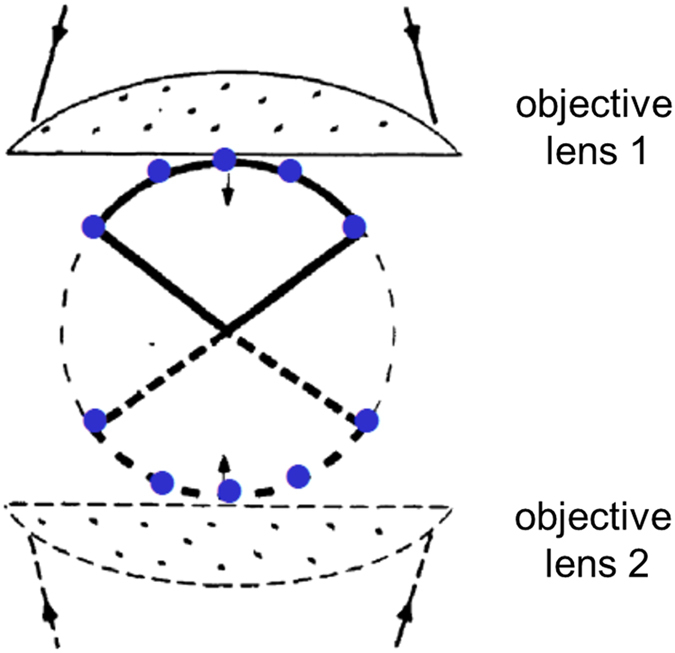
Schematic presentation of the arrangement used to calculate the focal intensity distribution in the case of “conventional” 4Pi microscopy. The drawing in black shows the case of “conventional” 4Pi microscopy, using two opposite high numerical aperture objective lenses^17^: In conventional far-field light microscopy only a segment of a spherical wave front is focused into or collected from an object point (indicated by the bold line, objective 1). A higher spatial resolution is achieved when not only a segment but a complete spherical wavefront is used (full 4π solid angle, broken thin line). However, focusing or collecting is already improved substantially (along the optical axis) when a second lens provides another segment (broken bold line) of the complete spherical wave front thus increasing the angles of the focused and/or collected wave. The blue “dots” schematically show the spatial distribution of the light sources S_i_ (Fig. 1) to simulate the conventional 4Pi case. The light sources with the largest angle towards the optical axis indicate the solid angle of the area containing the light sources with the collimated beams. Modified from Hell *et al*.^17^.

This vector approach of Feynman appeared to be particularly suited to calculate the focal intensity produced by an arrangement of multiple collimated beams as indicated in Fig. 10. On one hand, for an increasing number of beams, the results obtained by the calculation according to Feynman are expected to approximate the results calculated according to e.g. Richards and Wolf ^60^, thereby approximating the focal intensity distribution of a theoretical (aberration free) objective lens. On the other hand, for a large number of beams distributed over a very small solid angle, the results are expected to correspond to those obtained for low NA objective lenses, in which the spread of polarizations for the incoming light is very small, since depolarization of the incoming light by the low NA lens has no significant influence. In such a case, vectorial addition of the polarizations is not required, effectively rendering the problem to being of scalar nature.

The equations used throughout the manuscript to calculate the intensity distribution generated by multiple coherent light sources are described in Supplementary Note 3 for both the vector and the scalar/Feynman approach. The differences in the results for the two approaches are discussed in Supplementary Note 1. The collimated light sources in practice are taken to be Gaussian beams with the beam waist at the position of the nominal target site (the origin). Alternatively, other realistic representations of the beams such as e.g. Bessel beams could be used, as demonstrated successfully in the lattice light-sheet microscope developed in the Betzig lab^23^.

## Electronic supplementary material

Supplementary information
