# Supplementary material for: Super-resolution microscopy with very large working distance by means of distributed aperture illumination
Source: Sci Rep. 2017 Jun 16;7:3685. doi: 10.1038/s41598-017-03743-4 (PMC5473833; doi:10.1038/s41598-017-03743-4)
Supplement: Supplementary file 1 — Supplementary information [file 41598_2017_3743_MOESM1_ESM.pdf]

Supplementary Information

Super-resolution microscopy with very large working distance by means of distributed aperture illumination

Udo Birk[1,2,3], Johann v. Hase[1], and Christoph Cremer[1,2,3,4]

[1]Superresolution Microscopy, Institute of Molecular Biology (IMB), D-55128 Mainz, Germany;

[2]Physics Department University Mainz (JGU), D-55128 Mainz, Germany;

[3]Kirchhoff Institute for Physics, University Heidelberg, D-69120 Heidelberg, Germany;

[4]Institute of Pharmacy&Molecular Biotechnology (IPMB), University Heidelberg, D-69120 Heidelberg/Germany;

Corresponding author: e-mail: c.cremer@imb-mainz.de

Below we describe a number of steps relating to the actual setup of the distributed aperture microscope (DAM). These steps include: The errors made when using scalar calculation of the illumination intensity distribution; a realistic assumption about the minimum number of light sources required to set-up a functional DAM system; the formulas used in the actual (numerical) calculations; and a feasible experimental layout for implementing the DAM system, including pre-alignment procedures of the system and adjustments necessary during the actual data acquisition.

1. *Error due to use of scalar approach*

Table ST1. Extent of the central maximum of the illumination light distribution for 64 light sources, full 4Pi geometry. Excitation wavelength λ = 488, refractive index n = 1.518.

	FHWM-x (nm)	FHWM-z (nm)
linear polarization	135	171
circular polarization	151	151
scalar approximation	143	143

In most of the calculations, the scalar approach has been used for simplicity and speed of implementation. We investigated, to what degree the use of a scalar approach affects the accuracy of the calculations and the predicted outcome of the optical setup. We calculated the extent of the 4Pi focal volume (observation volume of the point-spread-function [PSF]) for three different cases: 1) linear polarization, 2) circular polarization, 3) scalar approximation. The results for the full-width at half-maximum (FWHM) in these three cases are summarized in Table ST1 when illuminating with 64 sources distributed homogeneously (compare Figures 1 and 7 of the main text) over the solid angle

4π. The differences between values observed in the FWHM when using scalar approximation and those observed using full vector description (electromagnetic waves) are well below the variations when using differently polarized incident light or different orientations of the polarization. Generally, the values obtained when using the scalar approximation provide a reasonably good estimation of the FWHM, considering that other factors such as an inhomogeneous refractive index and the mechanical stability (positioning, directional, and phase stability) also have an effect on the experimentally realizable minimal extent of the central illumination spot.

As an alternative application, when using the distributed aperture microscope setup as a replacement for confocal laser scanning microscopy illumination from a single side (see main text, Figure 5, for values obtained using ca. 6,500 sources), the following Table ST2 lists the values for the FWHM observed using electromagnetic wave (EM) description (row 1+2) and scalar approximation (row 3) for 57 sources. Dependent on the polarization and xyz directions, the differences between the FWHMs in a given direction varied between 2 nm and almost 70 nm. However, altogether these values are very similar to those experimentally obtained in a single high-NA objective lens microscope setup. Figure S1 provides the corresponding focal intensity distributions. While the focal plane intensity distribution shows a single high maximum and very low side lobes, the intensity distribution along the optical axis was found to contain a number of prominent side lobes.

Table ST2. Extent of the central maximum of the illumination light distribution approximating a single objective lens confocal laser scanning microscope using 57 light sources. Excitation wavelength λ = 488, refractive index n = 1.518. Illumination intensities are weighted according to the cosine of the angle α with respect to the optical axis, in order to approximate the transmission pupil function of a real objective lens.

	FHWM-x (nm)	FHWM-y (nm)	FHWM-z (nm)
linear polarization	169	235	483
circular polarization	197	197	483
scalar approximation	167	167	479

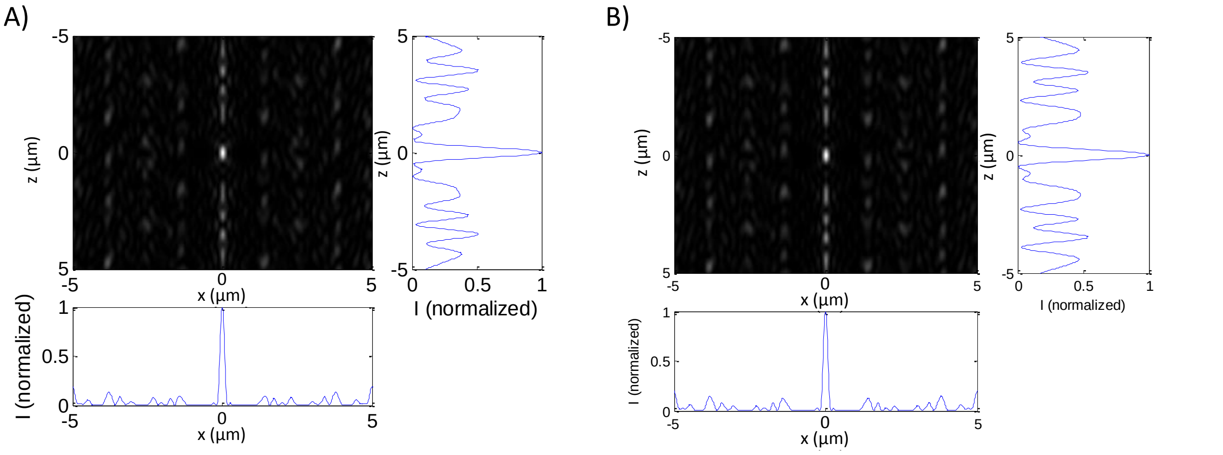

Figure S1. Sections across the central spot obtained when illuminating using 57 sources. A) Results when using full EM description. B) Results using scalar theory. As well as the differences in the numeric values (see **Table ST2**), also the overall shape and the extracted line profiles through the origin (along the x and the z axis) are very similar.

Note that in this case, the contrast in the images is reduced, as the intensity in the first minimum reaches up to ca. 1.7% of the central maximum (for circular polarization) or 0.95% for linear polarization.

2. *Number of light sources required*

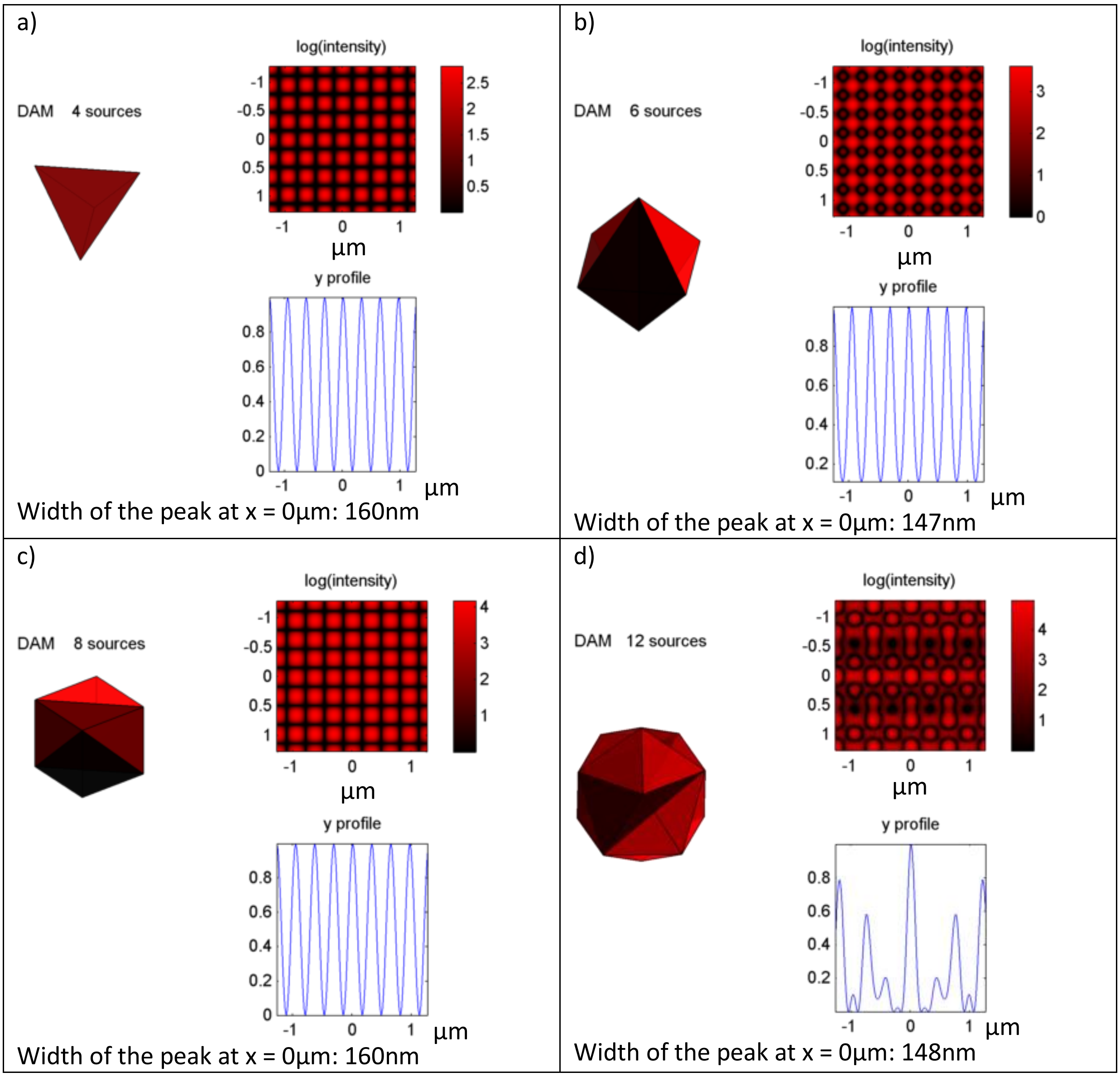

Figure S2. Extent of the central spot with increasing number of sources. Left: positioning of a number of sources on a spherical surface (source positions are at the end points of the edges). Right, top: calculated in-plane intensity distributions. The differences observed are due to a non-complete approximation of the spherical wavefront, and for the few sources used in the simulations strongly depend on the orientation of the coordinate system with respect to the light sources. Right, bottom: intensity profile across the center of the illuminated volume (x=z=0). Width of the peak is given as FWHM.

The number N of simultaneously irradiating, coherent light sources determines the width of the central maximum (the origin of the coordinate system) i.e., the focus in conventional focused excitation microscopy such as confocal laser scanning microscopy. In the ideal case, the following

extent of the central maximum was determined using simulations. The following Figure S2 shows the number and 3D arrangement of the few light sources (left) of the distributed aperture microscope (DAM) together with the in-plane intensity distribution (top right) and the profile across this intensity distribution at x=z=0 (bottom right) for a) 4 sources, b) 6 sources, c) 8 sources, and d) 12 sources. Other simulation parameters were an excitation wavelength of λ = 488 nm and a refraction index n = 1.518.

From the intensity distribution depicted in Figure S2d (FWHM of the central peak ca. 150 nm in xyz) it might be deduced that the corresponding illumination configuration – if combined with confocal detection at high NA (i.e. 200µm working distance) – provides a resolution in terms of 3D volume of the central spot[1,2] similar to 2-Photon 4Pi microscopy[3]. A big advantage of the DAM implementation is the use of 1-Photon excitation (and hence the feasibility of low illumination intensities) which is an important step towards possible *in vivo* time lapse observation at a resolution corresponding to that of a 4Pi 2-Photon microscope.

A remarkable outcome of this study is that for N >= 34 sources, the FWHM of the 'focus' is approximately constant (at ca. 143nm using the scalar approximation), which means that in principle a well-defined i.e. 3D minimized spot size can be obtained with as little as 34 individual light sources.

3. *Calculation of the intensity distribution*

In the following, we assume that an original, single, coherent laser light source is split into several beamlets, and that each of the beamlets is used as an individual source to illuminate the sample from various directions. According to Richard Feynman[4], instead of waves, light propagation is considered as a suitably defined flow of photon 'particles'. In order to mathematically express the propagation, a set of equations is required which can be used to describe the polarization and directionality of the photon flux, as well as its strength at every location (and hence its extent). As in our practical approach each of the beams has a limited divergence over the volume of interest, we use a local coordinate system with the optical axis along the center of the beamlet, and apply the same formulae which are available for wave optics (e.g. ABCD matrix formulation) to describe the effects of optical elements on the photon flux of each individual beamlet. In particular, in the optical setups described here we make use of Gaussian optics to describe the extent of the photon flux, and potentially to investigate the phase delay of the photon flux over the plane perpendicular to the direction of propagation.

Each individual source $i = 1,..,n$ contributes by a vector A_i to the light distribution at any site (x,y,z). The vector $A_i(x,y,z)$ for each source is calculated in the following way:

Let $A_{i,0}$ be the vector describing a light source directed parallel to the z-axis at the position of the light source i. The same light source directed towards the origin (0,0,0) can be obtained by rotating the polarization direction according to the direction of propagation (i.e. the negative of the source position)

$$\begin{pmatrix} \cos(\vartheta) & \sin(\vartheta) & 0 \\ -\sin(\vartheta) & \cos(\vartheta) & 0 \\ 0 & 0 & 1 \end{pmatrix} \begin{pmatrix} \cos(\alpha) & 0 & -\sin(\alpha) \\ 0 & 1 & 0 \\ \sin(\alpha) & 0 & \cos(\alpha) \end{pmatrix} \begin{pmatrix} \cos(\vartheta) & -\sin(\vartheta) & 0 \\ \sin(\vartheta) & \cos(\vartheta) & 0 \\ 0 & 0 & 1 \end{pmatrix} \overrightarrow{p_0} \qquad (1)$$

where $\overrightarrow{p_0}$ is the direction of polarization of the original laser light source, α and ϑ are the elevation and the azimuthal coordinate of the source i (beamlet) projected onto the unit sphere (see Figure S3).

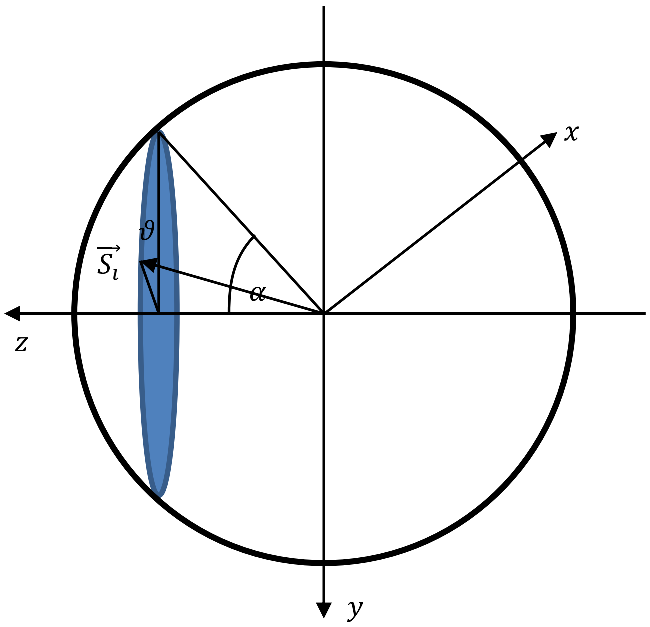

Figure S3. Coordinate system (unit sphere). The position of each source $\overrightarrow{S_\iota}$ is obtained by a combination of rotations around the y- and the z-axis.

The real, physical light source is of course not a point source, but is extended. In practice, we take as light source the exit pupil of the focusing optics used to obtain the collimated beam. In the following, we assume that the beamlet from each source is collimated at the origin. We can now describe the 3D light field (photon flux) from each beamlet by knowing the polarization and extent of the source at the exit pupil by Gaussian beam optics.

In this case, the beam diameter at the origin (beam waist) is given[5] by

$$2\,\omega_0 = \left(\frac{4\lambda}{\pi}\right)\left(\frac{f}{D}\right) \tag{2}$$

and the DOF (Depth of Field) is given by

$$\mathrm{DOF} = \left(\frac{8\lambda}{\pi}\right)\left(\frac{f}{D}\right)^2 \tag{3}$$

where f is the focal length of the focusing optics using to collimate the laser beam, and D is the diameter illuminated on the focusing element (e.g. the diameter of the laser beam at the position of the lens).

At a distance z from the origin, the beam diameter is ω can be obtained from the equation

$$\omega^2(z) = \omega_0^2\left[1 + \left(\frac{\lambda z}{\pi\omega_0^2}\right)^2\right] \tag{4}$$

and the wave-front curvature is given by

$$R(z) = z\left[1 + \left(\frac{\pi\omega_0^2}{\lambda z}\right)^2\right] \tag{5}$$

The largest curvature (minimum radius) $R_{\min} = 2\pi\omega_0^2/\lambda$ is observed at a distance z equaling the Rayleigh length $z_{\mathrm{R}} = \frac{\pi\omega_0^2}{\lambda}$.

We are interested in the light distribution within a small distance of the origin only, such that the change in collimation is negligible. The (complex) light distribution at a given point (x,y,z) contributed by a single Gaussian beam in the vicinity of the origin can be written[6] as

$$E(r,z) = E_0 \frac{\omega_0}{\omega(z)} \exp\left(-\frac{r^2}{\omega(z)}\right) \exp\left[-ikz - i\frac{2\pi}{\lambda}\frac{r^2}{2R(z)} + i\varphi(z)\right] \quad (6)$$

where $r = \sqrt{x^2 + y^2}$ is the radial distance from the axis $z = 0$, and $\varphi(z) = \arctan(z/z_R)$ is the longitudinal phase delay at distance z.

Using the Feynman approach instead, polarization of the sources is neglected. The position-dependent phases of the photons are regarded to be the hands on a clock. The Euclidian length of the hands corresponds to the amplitude of the light wave. These phase-vectors are added component-wise.

In both cases (the scalar approach according to Feynman, and the vectorial approach including polarization), the complex light distribution of each beamlet in the vicinity of the origin is numerically calculated using a common coordinate system (i.e. a rotation of the beamlet coordinate system onto the common Cartesian coordinate system). The final distribution at the position (x,y,z) can be obtained as the vector-sum over all contributions from all sources at the position (x,y,z).

Similar results might be obtained when using Bessel beams instead of Gaussian beams. However, the extent of the central illumination spot can be shown to be larger when using Bessel beams. The benefit of using Bessel beams over Gaussian beams lies in the stability of the illumination pattern. The simultaneous use of multiple Bessel beams to arrive at a well-defined illumination pattern has been described previously in the literature.[7]

4. *A Possible Experimental Realization of the Setup*

4.1. Generation of collimated illumination light sources

In the following, we assume that each source (beamlet) is equipped with a suitable collimation optics, e.g. a diffractive optical element (DOE) or a small lens.

A. Using open beam (free space) illumination

When using an open beam optics illumination scheme, the width of the beams in the sample region together with the depth-of-field (DOF), in which the laser light is approximately collimated, can be deduced from Gaussian beam optics. Using an excitation wavelength of 488nm, we obtain for a 30mm working distance (focal length) of the illumination system numerical values for the beam waist of $2\,\omega_0 = 19\ \mu\mathrm{m}$ and for the depth-of-field of $\mathrm{DOF} = 1.12\ \mathrm{mm}$. These values indicate that slight aberrations are tolerable in respect to the direction of the beam, under the condition that the phase of each of the incident beams can be adjusted to yield constructive interference with all the other beams. Numerical simulations indicate that phase fluctuations corresponding to about $\lambda/4$ are tolerable, without significantly compromising the extent and contrast of the focal spot. However, with increasing phase fluctuations the

contrast of the illumination spot, i.e. the ratio between fluorescence excitation at the origin and excitation in neighboring regions (with supposedly no excitation) will rapidly decrease. As a consequence, the phases of the light sources need to be controlled by means of adaptive optics (see below). From the extent of the beam waist, we assume that a pointing stability of 100 mrad is required for each of the individual beams, which is easily maintained by most commercial laser light sources.

The phase delay at z_{R} due to the curved wavefront at maximum beam radius is given by

$$\frac{2\pi}{\lambda}\frac{\omega^2(z_R)}{2R_{\min}} = \frac{2\pi}{\lambda}\frac{2\omega_0^2\lambda}{4\pi\omega_0^2} = 1\ \mathrm{rad}$$

corresponding to $\lambda/(2\pi)$. As outlined above, the phase delay induced by the curved wavefronts of Gaussian beams does not contribute to a significant error in terms of broadening or contrast of the central spot.

B. Using fiber illumination

In this scenario we have to collimate light originating from a very small source (a fiber tip), as shown in Figure S4. The single source has a radius of y_1 and emits light under a maximum angle θ_1. If we collimate the output from this source using a focusing element (e.g. a lens) with focal length f, then the result will be a beam with a radius $y_2 = \varphi_1 f$ and divergence angle $\varphi_2 = y_1/f$.[8] It is clear that, no matter what focusing power is used, the beam radius and beam divergence have a reciprocal relation.

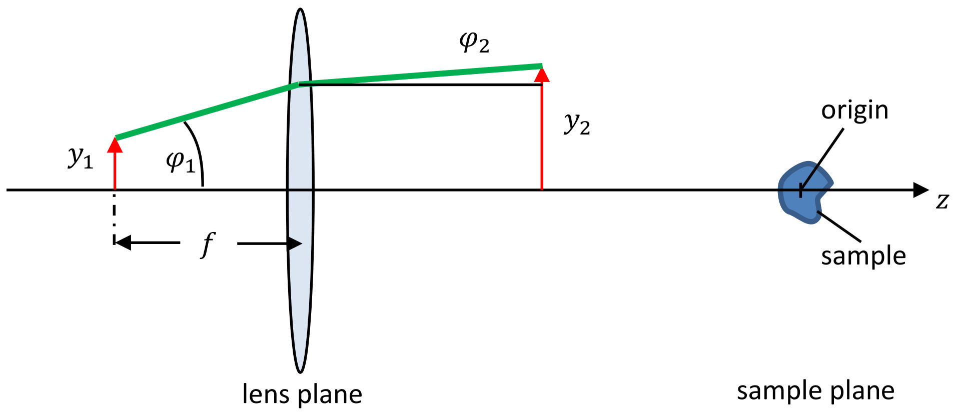

Figure S4. Collimation of light emitted from a fiber tip.

A typical polarization maintaining fiber for the 488 nm laser wavelength has a mode field diameter of ca. 4 µm and a numerical aperture (NA) of 0.13. The radius y_1 of our source is then 2 µm. NA is defined in terms of the half-angle accepted by the fiber, so φ_1 = 0.13. In this case we would use a 2 mm focal length lens to collimate the output (fiber-to-free space coupler), and arrive at a beam with a radius of 260 µm and a half-angle divergence of 1 mrad. A slight defocus of the coupling lens would result in the collimation plane being shifted from the lens plane to the sample plane, at the cost of a slightly larger beam diameter.

The phase delay due to the curved wavefronts of the Gaussian beams is likely to be smaller than for the open beam in 4.1.A, because of the larger beam waist.

4.2. Required working distances when using a large number of light sources

In the case of a fiber based illumination, the minimum area covered by each light source is given by $A_{\text{source}} = \pi(260\mu\text{m})^2 = 0.212$ mm². This area is compatible with the diameter of a typical single mode fiber including the cladding and the coating, which together is often in the 0.25mm diameter range. The fiber jacket would needs to be removed in this case. Depending on the distance R between the source and the origin, the solid angle covered by each source is given by

$$\Omega_{\text{source}} = \frac{A_{\text{source}}}{4\pi R^2}. \quad (7)$$

For instance at a illumination working distance of $R = 30$ mm, each fiber source covers a solid angle of $1.88 \cdot 10^{-5}$, or roughly 0.24% of the full solid angle. At a primitive (simple) 2D packing (packing density $\frac{\pi}{4} \approx 78.5\%$) this working distance would allow us to place $100\%/0.24\% * 0.785 \approx 3328$ sources around the object. In order to place 10,000 sources around the object, an illumination working distance of $R = 52$ mm would be required. We might consider placing the additional collimation optics in front of the fiber including the jacket, thereby increasing the actual fiber diameter to ca. 3mm. In this case, the working distance of 30 mm would allow us to place ca. 100 sources at a primitive packing ratio, which is more than sufficient for the illumination scheme using 57 fiber sources outlined below.

4.3. Sample mounting and detection of fluorescence signal

Several options exist for the spatial arrangement of the sample holder and the optical components used for detection of fluorescence emitted by the sample. A simple, but effective method for detection of fluorescence light is to use a long working-distance objective lens. As the focal volume in which fluorescence is generated is defined by the quality of the illumination light distribution, the important quantity for the detection light path is the amount of light collected rather than to obtain an aberration-free imaging system. For instance, the use of a cemented doublet, achromatic condenser lens or even an aspherical, achromatic doublet is likely to give higher detection efficiency as compared to a long working distance objective lens system, with both the f-number and the numerical aperture being large in order to have large working distances combined with high detection efficiency. (The lack of correction for higher order aberrations requires this approach to be combined with object scanning rather than beam scanning). However, unlike in a 2-photon microscope system where the signal is detected from a volume solely defined by the excitation spot, the typically missing pinhole of the DAM system at very large working distances will render out-of-focus excitation to contribute to the level of the background, if the focus of the detection optics is not well-defined. Such relatively simple condenser lenses can be made with much larger diameters as compared to high-end objective lenses, allowing e.g. imaging with a numerical aperture of NA = 0.53 (NA = 0.76) at a working distance of 31mm for aspheric doublets (singlets). (See e.g. catalogues from Edmund optics, Thorlabs, Ulooptics, Newport, etc.). The aspheric doublets naturally have much better control of the focal position and extent over the range of wavelengths emitted by the fluorophores.

An alternative solution is to use long working distance objective lenses, which offer very good corrections for focal position, and various forms of image distortions. For working distances of 20mm, objective lenses with numerical apertures of up to 0.42 are available (e.g. Mitutoyo Plan Apochromat). A benefit of using objective lenses is that, similar to the 4Pi Type B/C microscope detection[2,9], in principle several of these lenses can be combined to arrive at a much larger

composite synthetic aperture, provided that the objective lenses can be aligned to better than approx. 30nm, and that the dispersion is corrected in the individual detection beam paths. This might be possible to achieve in a lab-based instrument, but will be difficult to implement in a commercialized version of the setup.

Finally, a long working distance objective lens with a large field of view has recently been brought forward by Brad Amos and colleagues[10]. They termed their instrument Mesolens Confocal, and the instrument has been shown to facilitate imaging at large depths with high quality. However, their system has not been shown to operate in one of the recently developed super-resolution imaging modes based on objective lenses, any of which can be implemented using the DAM illumination scheme (see main text). We would therefore propose the use of a device similar to the Mesolens developed by Amos et al. as an alternative solution for the detection of fluorescence signal in the DAM system, possibly optimized towards even higher working distances. Using such a detection configuration, aberrations in the detection can be largely minimized.

As discussed in the main text, the detection efficiency scales with NA^2 i.e., for a detection system using an NA of 0.44, the number of photons detected would be $(0.44/1.4)^2=0.1$. This means that only 10% of the signal which we would detect using a 1.4 NA objective lens would be collected by each of the detection lenses. In single molecule localization microscopy applications, this means a three times worse localization precision and hence optical resolution[11]. In many cases, however, this disadvantage may be overcome in many cases by appropriate specimen preparation.

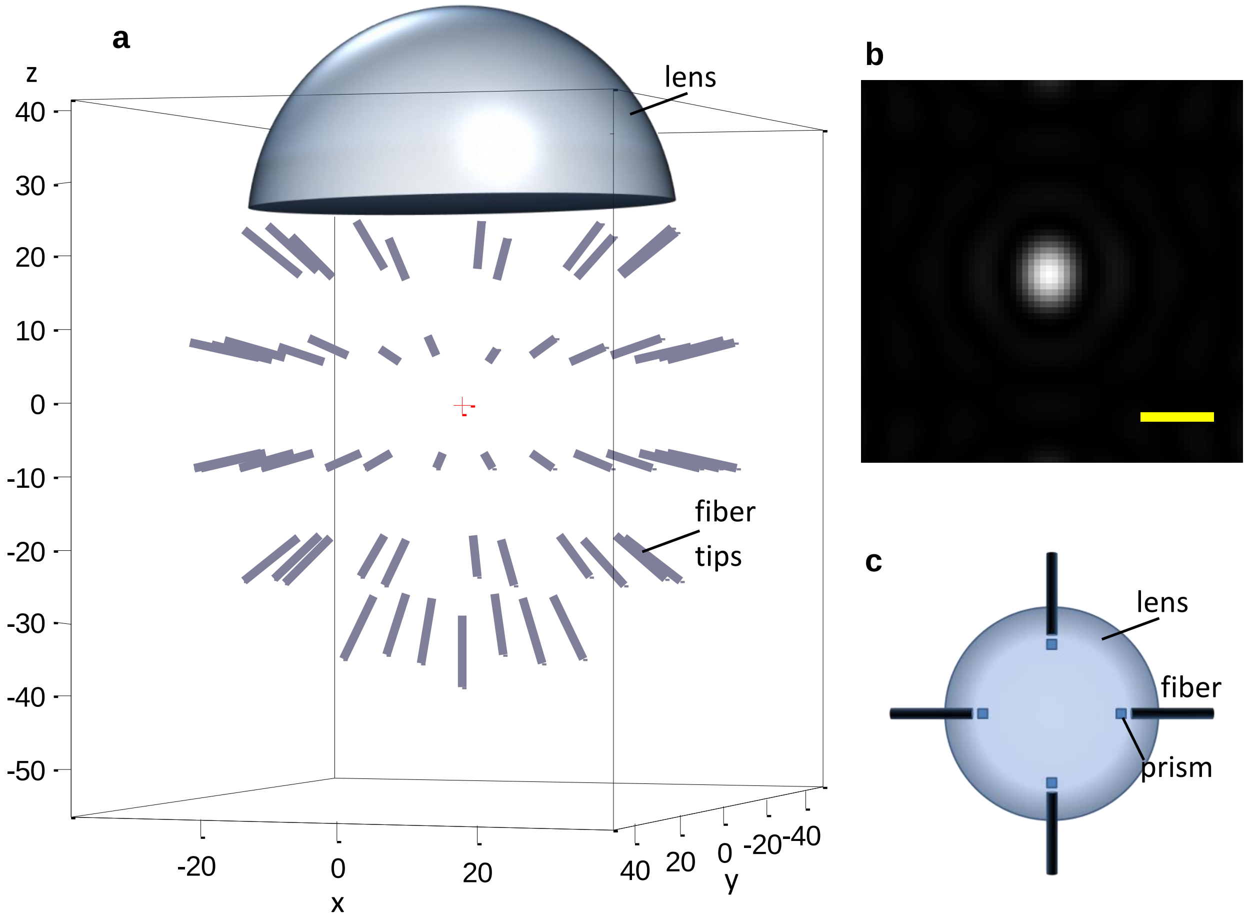

Figure S5. Illumination using 57 fibers. a) 3D rendering of the illumination of the sample with fiber tips with part of the full 4π solid angle being left out for the detection lens (top). The fiber tips are equipped with appropriate collimation optics. The axis dimensions are in mm. The distance between fiber ends and geometrical center (red '+', sample position) is 30 mm, the working distance of the detection lens (aspheric doublet) is 31 mm. b) Intensity distribution of the central spot when using the illumination scheme in a). As expected from the non-symmetric illumination, the shape is slightly elongated at an FWHM of ca. 215/260nm in x/z when imaging in air (not shown), and 140/171nm in x/z when imaging in oil (n = 1.518). Scale bar: 200 nm. c) Scheme for a true 4π illumination configuration (bottom view). The sources (fiber tips) which are missing because of the detection lens are introduced from the side in the detection cone (in front of the detection lens) using a set of prisms attached to the fiber tips. This way, the light can be deflected towards the origin.

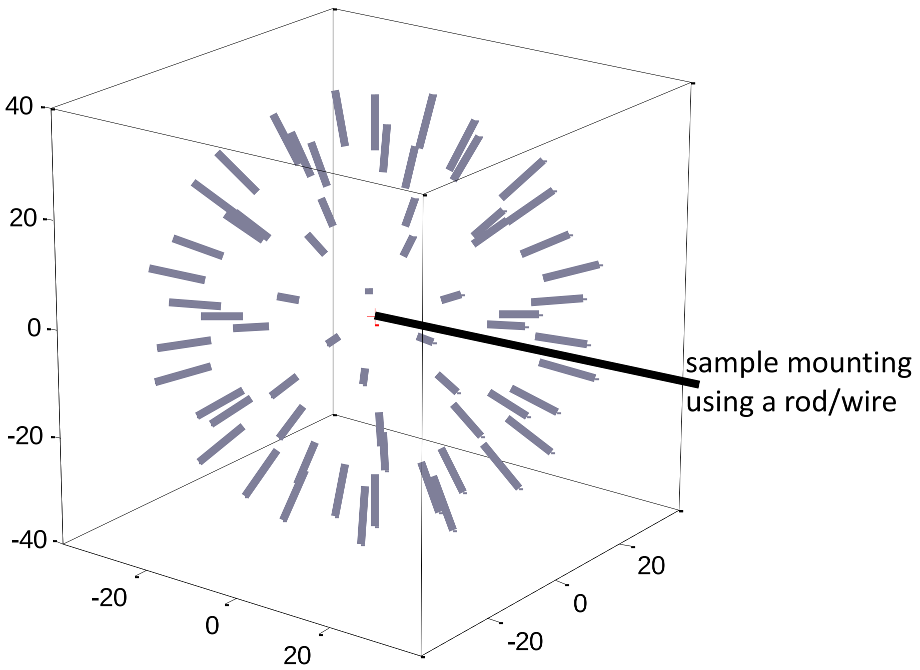

Figure S6. Illumination using 64 fibers. Illumination of the sample with fiber tips leaves enough space for additional components for holding the sample, e.g. via a blackened metal rod or wire. In this case, the fiber tips were assumed to have a diameter of ca. 2mm. The wire will lead to a distortion of the illumination field; however, these distortions are negligible if the wire is thin. The axis dimensions are in mm. The distance between fiber ends and geometrical center (red '+', sample position) is 30 mm. Best stability is likely to be obtained in the case when the sample mounting is introduced from the bottom (the sample rests on the tip of the rod), and the detection is from the top.

For lens based detection at large working distances, an NA of 0.5 might be realized, indicating an optical resolution (detection side) of about $d = 0.61\ \lambda/\mathrm{NA}$ ≈600 nm. For illumination with a few sources only (e.g. 12 sources), considerable signal would be detected from the side lobes around the central peak. However, such neighboring maxima might be rejected by introducing a pinhole in the detection path just before the detector. The pinhole size should not be smaller than 1 Airy Unit, in order not to reduce the already weak signal (due to the lower NA = 0.5). The resulting geometrical confocality has the additional benefit of effectively suppressing signals originating from regions which are not in focus of the detection lens.

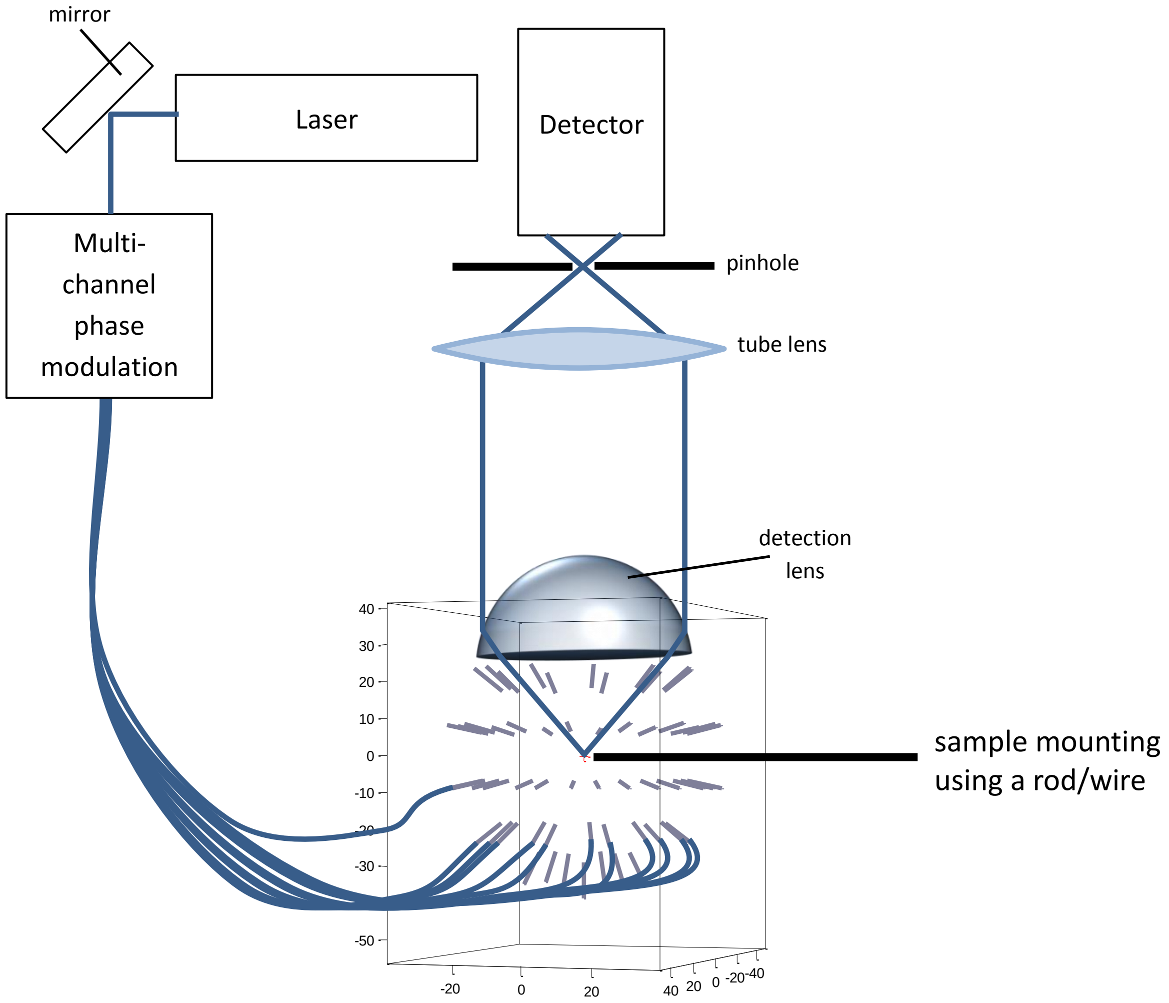

Figure S7. Illumination and detection scheme (using 57 fibers). Control of the phases of the illumination beams is obtained by phase modulation of the 57 sources, e.g. by means of 57 individual phase modulators, or more cost effectively, by a liquid crystal device (i.e. a spatial light modulator). For clarity, not all 57 fibers have been drawn.

4.4. Positioning of light sources

We will discuss in the following two main scenarios for distributing the light sources around the sample. Option 1 is based on an open beam configuration which involves a number of mirrors and focusing lenses for each of the individual beams to be placed around the sample. This option has the advantage that it provides highest modularity, but is rendered impractical for more than a couple of individual beams (e.g. 10 to 20) due to the time needed to adjust position, direction, polarization, and phase of each individual illumination beam (see Figure S8). Alternatively, a fiber optics approach can be realized in which the fiber ends are acting as the individual light sources, and the fiber ends are organized in such a way that they all end on a spherical surface around the origin, representing the ideal far-field spherical wave.

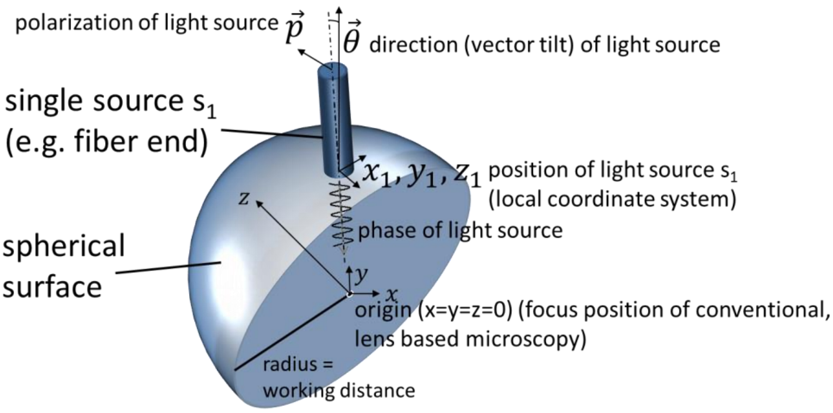

Figure S8. Positioning of the light sources.

Alignment of the individual x_i, y_i, z_i can be achieved by precise machining of a sphere (or part of a sphere) with holes for the respective optical elements. A similar mounting of individual transducer elements on a spherical surface has been realized in the case of photoacoustic detection of breast cancer[12].

4.5. Alignment of source positions on the spherical surface

The positioning of the sources on the spherical surface, or more precisely, a lateral offset of the sources from the design position has little effect on the extent of the central spot size. Simulations indicate that the width of the central spot is not significantly affected if the individual sources are offset by a fraction of their mutual distances. Much more critical is the alignment and stability of the phases of the individual light sources, as discussed in the next two subsections.

4.6. Adjustment of phases

From our experience, the long term stability of the alignment is an issue in setting up a multi-beam illumination and/or detection scheme. Usually, the adjustment depends critically on the thermal expansion coefficients of the materials used to mount the optical components in the illumination/detection light path, and on the temperature stability of the system that can be maintained by external devices such as e.g. a temperature stabilized laminar flow box. Based on our previous work[13], we suggest a two stage phase alignment for the implementation of the system. The phases of the illumination beams are individually optimized by adjusting the path length e.g. using electro-optical phase modulators. At ca. 2000 EUR per electro-optical element, a simpler mechanical solution in which the phases are modulated e.g. by adjusting the beam path (for instance using a gap between two optical elements) will be much more cost-effective. This adjustment of the phase of each individual beam is very time consuming, and therefore needs to be facilitated by an automated fine adjustment of the phases prior to the actual measurement. Principally, the pre-adjustment described in this subsection and the fine adjustment described in Section 4.7 can be done using the same alignment protocol.

Naturally, both the coarse pre-adjustment of the instrument and the fine adjustment prior to the actual measurement need to take into consideration that the sample itself having non-homogeneous refractive index introduces phase shifts to the incident beamlets, resulting in both tilting and phase delay of the impingent Gaussian wavefronts. The adjustment of the phases can be accomplished in an incremental alignment procedure, in which always two sources on opposite sides of the sample are adjusted to yield a standing wave with the wavefronts being perpendicular to the common axis (This might require a detection scheme in which the sample can be observed from different sides, e.g. by moving the detection lens with respect to the common axis of the two beamlets). Next, the phase delay in one of the beams is modified to yield maximum intensity at the origin. Subsequently, the next set of two beamlets (featuring an angle with respect to the common axis of the first set of two beamlets) is adjusted in a similar manner, i.e. first aligning the common axis to yield the perfectly aligned standing wavefield, and next adjusting the phases to yield maximum intensity of this second standing wavefield at the origin. The illumination intensity of the previously adjusted beams will need to be reduced for adjustment of the further beamlets, in order to maintain sensitivity of the procedure. It should be noted, that due to the point-scanning realization of the setup, precise alignment of the standing wave axis is not strictly necessary, but it would be beneficial for the system characterization and it would allow much more precise control of the phases, especially during the interpolation step.

The alignment procedure will require suitable calibration objects. Such calibration objects can be obtained e.g. in the form of fluorescent nanospheres or semiconductor quantum dots, both of which provide a very stable output signal.

4.7. Operation

As described above in the section on detection of the fluorescence signal, the proposed layout of the instrument presently assumes a stage scanning configuration. Consequently, the data acquisition process is likely to be longer compared to those of present beam scanning microscope systems.

As the sample itself introduces phase aberrations due to the varying refractive index of the non-homogenous sample, additional calibration objects will need to be prepared together with the sample, and the phase adjustment at these reference points needs to be monitored In order to be able to interpolate the phase adjustments necessary in-between these reference points. Conceptually, this is very similar to how multi-color acquisitions are registered today, although in the case of DAM acquisitions, a registration in a post-acquisition step is not possible, and the phases must be adjusted live during the data acquisition.

A convenient way to achieve fast phase adjustment is to use a spatial light modulator. The light output for individual pixels (or subsets of pixels) are injected into a single polarization maintaining fiber. In this way, the phases of each of the beamlets can be adjusted simultaneously. In contrast to present adaptive optics (AO) systems, only the phases of the wavefronts can be adjusted; tilting of the wavefronts is not possible in this configuration. However, due to the fact that the spherical wavefront of the incident (conventional) 4Pi microscope is approximated by a discrete set of individual beamlets, the net effect of the spatial light modulator together with the large number of beamlets is similar to active compensation of wavefront aberrations in an AO system. In fact, a number of commercial AO systems make use

of a similar discretized approach to correct the wavefront aberrations in a microscope system. Also the implementation of the feedback loop in the DAM system is not as straight forward as it is in an AO system. Direct monitoring of the wavefronts is not easily possible, therefore the phase adjustments necessary to obtain a small illumination volume need to be determined at a number discrete sites throughout the sample, requiring the aforementioned calibration objects to be prepared together with the actual sample. For each sample, the pre-acquisition step is therefore to record the phase-adjustment at the calibration sites, and then start the acquisition interpolating the phase changes in-between the calibration sites.

5. Minimal STED depletion donut using DAM

As shown in Figure 9 (main text), in principle the DAM setup allows to generate a STED depletion beam with customizable mode. As shown by Chmyrov et al.[14], it is possible to superimpose two standing wave fields with orthogonal polarization to generate a 2D illumination pattern with a large number of minima, in which the intensity drops to truly zero. In a stage scanning approach suggested here, a large number of minima (donuts) is not required. However, a similar approach as outlined by Chmyrov et al. could be used to superimpose 3 standing wave patterns, each standing wave pattern being generated by illuminating the sample using two sources having linear polarization along the same axis. If the three standing wave patterns are oriented along the three coordinate axes, the polarization of each of the three standing wave patterns can be chosen to have no overlap with the other two standing wave patterns (see **Figure S9**). If scattering in the samples can be made negligible, for each of the standing wave fields, the intensities and phase differences in the two counter-propagating beams can be adjusted to yield zero intensity in the origin. As the polarization of each of the standing wave pattern is perpendicular to the other two, there will be no interference between the standing wave patterns, and consequently, it is relatively easy to obtain a high quality STED donut.

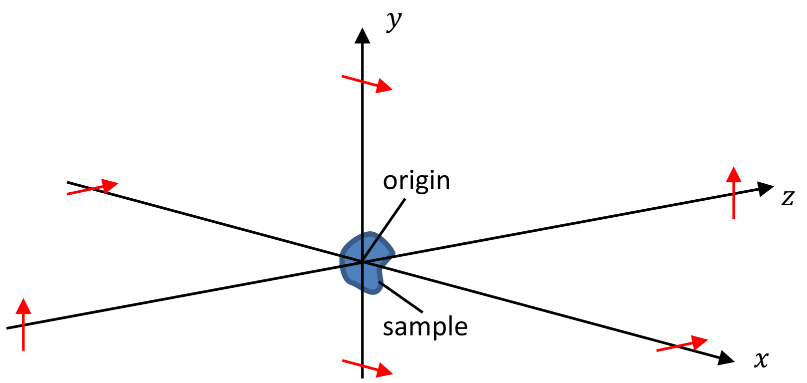

Figure S9. Generation of a STED 3D donut.

6. References

1. Hell, S. & Stelzer, E. H. Fundamental improvement of resolution with a 4Pi-confocal fluorescence microscope using two-photon excitation. *Opt. Commun.* **93,** 277–282 (1992).
2. Hell, S. & Stelzer, E. H. Properties of a 4Pi confocal fluorescence microscope. *J Opt Soc Am A* **9,** 2159–2166 (1992).
3. Hänninen, P. E., Hell, S. W., Salo, J., Soini, E. & Cremer, C. Two-photon excitation 4Pi confocal microscope: Enhanced axial resolution microscope for biological research. *Appl Phys Lett* **66,** 1698–1700 (1995).
4. Feynman, R. *QED: The Strange Theory of Light and Matter*. (Princeton University Press, 2006).
5. Meschede, D. *Optik, Licht und Laser*. (B.G. Teubner, 1999).
6. Wen, J. J. & Breazeale, M. A. A diffraction beam field expressed as the superposition of Gaussian beams. *J. Acoust. Soc. Am.* **83,** 1752–1756 (1988).
7. Chen, B.-C. *et al.* Lattice light-sheet microscopy: Imaging molecules to embryos at high spatiotemporal resolution. *Science* **346,** 1257998 (2014).
8. Newport Technical Notes. Available at: https://www.newport.com/resourceListing/technical-notes. (Accessed: 26th February 2017)
9. Hell, S. W., Lindek, S., Cremer, C. & Stelzer, E. H. Confocal microscopy with an increased detection aperture: type-B 4Pi confocal microscopy. *Opt. Lett.* **19,** 222–224 (1994).
10. Saini, A. New lens offers scientist a brighter outlook. *Science* **335,** 1562–1563 (2012).
11. Edelmann, P. & Cremer, C. in *Optical Diagnostics of Living Cells III, Proc. SPIE* **3921,** 313–320 (2000).
12. Jose, J., Manohar, S., Kolkman, R. G. M., Steenbergen, W. & van Leeuwen, T. G. Imaging of tumor vasculature using Twente photoacoustic systems. *J. Biophotonics* **2,** 701–717 (2009).
13. Baddeley, D., Batram, C., Weiland, Y., Cremer, C. & Birk, U. J. Nanostructure analysis using spatially modulated illumination microscopy. *Nat Protoc.* **2,** 2640–2646 (2007).

14. Chmyrov, A. *et al.* Nanoscopy with more than 100,000 'doughnuts'. *Nat. Methods* **10,** 737–740 (2013).
